# ATM phosphorylates the FATC domain of DNA-PK_cs_ at threonine 4102 to promote non-homologous end joining

**DOI:** 10.1093/nar/gkad505

**Published:** 2023-06-13

**Authors:** Huiming Lu, Qin Zhang, Daniel J Laverty, Andrew C Puncheon, Mathew M Augustine, Gareth J Williams, Zachary D Nagel, Benjamin P C Chen, Anthony J Davis

**Affiliations:** Department of Radiation Oncology, UT Southwestern Medical Center, Dallas, TX75390, USA; Department of Radiation Oncology, UT Southwestern Medical Center, Dallas, TX75390, USA; Department of Environmental Health, Harvard T.H. Chan School of Public Health, Boston, MA02115, USA; Department of Radiation Oncology, UT Southwestern Medical Center, Dallas, TX75390, USA; Division of Surgical Oncology, Department of Surgery, UT Southwestern Medical Center, Dallas, TX75390, USA; Department of Surgery, North Texas VA Medical Center, Dallas, TX75216, USA; Department of Biochemistry and Molecular Biology, Robson DNA Science Centre, Charbonneau Cancer Institute, Cumming School of Medicine, University of Calgary, Calgary, AB, Canada; Department of Environmental Health, Harvard T.H. Chan School of Public Health, Boston, MA02115, USA; Department of Radiation Oncology, UT Southwestern Medical Center, Dallas, TX75390, USA; Department of Radiation Oncology, UT Southwestern Medical Center, Dallas, TX75390, USA

## Abstract

Ataxia-telangiectasia mutated (ATM) drives the DNA damage response via modulation of multiple signal transduction and DNA repair pathways. Previously, ATM activity was implicated in promoting the non-homologous end joining (NHEJ) pathway to repair a subset of DNA double-stranded breaks (DSBs), but how ATM performs this function is still unclear. In this study, we identified that ATM phosphorylates the DNA-dependent protein kinase catalytic subunit (DNA-PK_cs_), a core NHEJ factor, at its extreme C-terminus at threonine 4102 (T4102) in response to DSBs. Ablating phosphorylation at T4102 attenuates DNA-PK_cs_ kinase activity and this destabilizes the interaction between DNA-PK_cs_ and the Ku-DNA complex, resulting in decreased assembly and stabilization of the NHEJ machinery at DSBs. Phosphorylation at T4102 promotes NHEJ, radioresistance, and increases genomic stability following DSB induction. Collectively, these findings establish a key role for ATM in NHEJ-dependent repair of DSBs through positive regulation of DNA-PK_cs_.

## Graphical Abstract

**Graphical Abstract:**
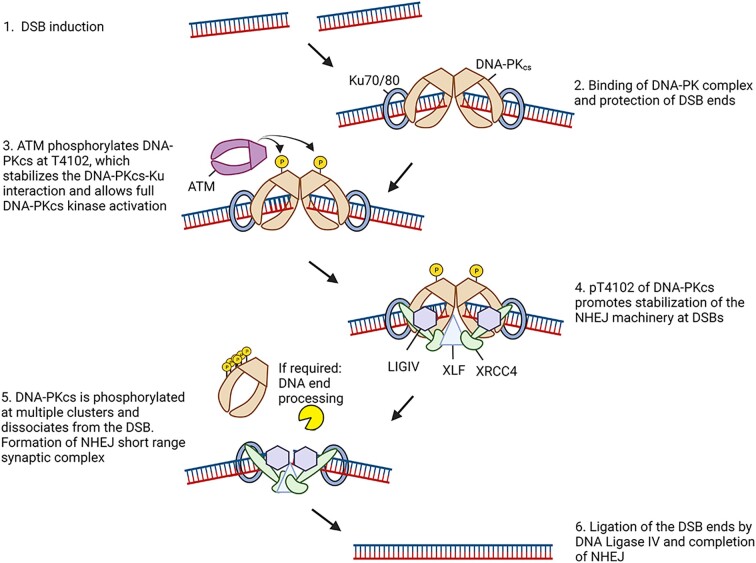
Phosphorylation of DNA-PKcs at T4102 by ATM promotes NHEJ-mediated DSB repair.

## INTRODUCTION

DNA double-stranded breaks (DSBs) are cytotoxic DNA lesions that pose an immediate threat to genome stability, and failure to properly repair them can lead to cell death, chromosomal aberrations, or carcinogenesis (1). Complex mechanisms collectively termed the DNA damage response (DDR) have evolved in cells to manage DSBs. These mechanisms include DNA damage recognition, activation of signaling cascades and cell cycle checkpoints, chromatin remodeling, transcription regulation, and repair of the DSB. Two members of the phosphatidylinositol-3-kinase-like kinase (PIKK) family, DNA-PK_cs_ (DNA-dependent protein kinase catalytic subunit) and ATM (ataxia telangiectasia-mutated), are instrumental in modulating the cellular response to DSBs (2). DNA-PK_cs_ and ATM are recruited to the site of the DNA damage by the sensors Ku (Ku70 and Ku80 heterodimer) and MRN (MRE11-RAD50-NBS1), respectively, which results in activation of each kinase's catalytic activity (3). Once activated, DNA-PK_cs_ and ATM phosphorylate substrates that drive multiple pathways responsible for the DDR, including the repair of DSBs.

DNA-PK_cs_ promotes DSB repair via the non-homologous end joining (NHEJ) pathway (4). In mammalian cells, NHEJ is the pathway primarily responsible for the repair of radiation-induced DSBs and DSBs generated for V(D)J and class switch recombination (CSR) during T- and B-cell lymphocyte maturation. NHEJ initiates when Ku rapidly binds directly to the DSB, where it then performs its primary function as a scaffold to recruit the NHEJ machinery to the damage site. It is still unclear if the recruitment of the NHEJ factors occurs in a stepwise sequential manner or via a dynamic assembly, but early studies found that the core NHEJ factors, Ku, DNA-PK_cs_, X-Ray Repair Cross Complementing 4 (XRCC4), DNA Ligase 4 (LIG4), and XRCC4-like factor (XLF) collectively stabilize at the DNA damage site via a number of protein-protein interactions and are modulated by DNA-PK_cs_ kinase activity (5). Single-molecule FRET studies revealed that NHEJ forms two synaptic complexes, referred to as ‘long-range’ and ‘short-range’ (6). The long-range complex synapses two DSB ends but they are more than 100 Å apart, whereas the DNA ends are more closely aligned in the short-range synaptic complex. DNA-PK_cs_ kinase activity was found to be important for the transition from the long-range to the short-range complex. Single-particle cryo-electron microscopy recently offered insight into the factors that may mediate the NHEJ synaptic complexes (7). The long-range complex contained the Ku heterodimer, DNA-PK_cs_, LIG4, XRCC4, and XLF. In the absence of DNA-PK_cs_, Ku, LIG4, XRCC4 and XLF formed a complex in which the DNA ends are horizontal and aligned for processing and ligation, similar to what is predicted for the short-range synaptic complex. Furthermore, evidence suggests that DNA-PK_cs_ activity promotes the processing of unligatable DNA ends by regulating the recruitment and stabilization of DNA end processing factors at DSBs and via autophosphorylation-induced conformational changes that free the DSB ends (4,8–10). However, there is still significant debate about the role and function of DNA-PK_cs_, DNA-PK_cs_ activity and DNA-PK_cs_ phosphorylation status in synaptic complex formation and transition (11).

ATM, the protein defective in the heritable disorder ataxia telangiectasia (A-T), is the kinase primarily responsible for signal transduction and cell cycle regulation in response to DSBs (12). Cells derived from A-T patients are hypersensitive to DSB generating agents, suggesting that ATM is essential for the repair of DSBs. ATM does not play a direct role in DSB repair but influences multiple pathways via phosphorylation events. First, ATM promotes homologous recombination (HR) by phosphorylating multiple HR factors, including MRE11, NBS1, PALB2, BRCA1, and CtIP (13–17). Second, ATM is required for NHEJ at complex lesions, DSBs induced in heterochromatin, and DSBs harboring a blocked DNA end (18,19). Moreover, ATM phosphorylates many components of the NHEJ pathway, such as XLF, Artemis, and DNA-PK_cs_, but there is limited knowledge of the functionality of these phosphorylation events (20). ATM plays at least one important additional role in NHEJ that has not been elucidated, but it is redundant with the core NHEJ factor XLF and thus not normally apparent (21). Finally, it has been reported that ATM is dispensable for NHEJ but promotes repair fidelity via an undefined mechanism (22).

Here, we uncover a novel function for ATM in NHEJ via phosphorylating DNA-PK_cs_. The data show that DNA-PK_cs_ is phosphorylated by ATM in its FATC domain at threonine 4102 in response to DSBs. Ablating phosphorylation at T4102 decreased DNA-PK_cs_ kinase activity and results in destabilization of the interaction between DNA-PK_cs_ and the Ku-DNA complex. Phosphorylation of DNA-PK_cs_ at threonine 4102 results in stabilization of the NHEJ machinery at DSBs, leading to increased NHEJ efficiency and genomic instability. Our results establish a model in which phosphorylation of the FATC domain of DNA-PK_cs_ by ATM promotes efficient NHEJ.

## MATERIALS AND METHODS

### Cell lines, cell culture, inhibitor treatments and cell cycle synchronization

Chinese Hamster Ovary (CHO) V3 cells and V3 cells stably expressing DNA-PK_cs_ wild-type, T4102A, and T4102D were cultured in Hyclone α-minimum Eagle's medium supplemented with 5% newborn calf serum, 5% fetal bovine serum (Corning), and 1X penicillin/streptomycin (Gibco). U2OS cells were cultured in Dulbecco's modified Eagle's medium supplemented with 10% fetal bovine serum and 1× penicillin/streptomycin (Gibco). All cells were grown in an atmosphere of 5% CO_2_ at 37°C. To inhibit DNA-PK_cs_, ATM or ATR, cells were incubated for 2 h prior to irradiation with 5 μM NU7441 (SelleckChem), 5 μM KU55933 (SelleckChem) or 5 μM VE821 (SelleckChem), respectively. Enrichment of U2OS cells at G1 phase and S/G2 were performed via a double thymidine block as previously described (23).

### Irradiation

Cells were irradiated with γ-rays generated by a Mark 1 ^137^Cs irradiator (J.L. Shepherd and Associates) with a dose of 10 Gy, unless otherwise indicated in the figure.

### Generation of antibody against phosphorylated DNA-PK_cs_ at T4102

Anti-pT4102 polyclonal antibodies were generated by immunizing New Zealand white rabbits with KLH (keyhole limpet hemacyanin)-conjugated phosphopeptide GLSEET[PO_3_]QVKCLMC. The phospho-specific antibodies were first passed through the corresponding unphosphorylated peptide-conjugated Sepharose CL-4B column (Pierce) to deplete IgGs that were not phospho-specific. The flow through IgGs were then affinity-purified using a phospho-peptide column. Eluted anti-pT4102 polyclonal antibodies were verified with phospho and non-phospho peptides via dot plot analysis.

### Site-directed point mutagenesis

Site-directed mutagenesis was performed using PCR to substitute T4102 on DNA-PK_cs_ with alanine or aspartic acid using the pCMV-F2-K and pCMV-YFP-K as a template to generate mutated FLAG-tagged and YFP-tagged DNA-PK_cs_, respectively. The primers used were: T4102A: T4102A Forward 5′- TGGGCTTTCAGAAGAGGCTCAAGTGAAGTGCCT-3′ and T4102A Reverse 5′-AGGCACTTCACTTGAGCCTCTTCTGAAAGCCCA-3′ and T4102D: T4102D Forward 5′-GTGGGCTTTCAGAAGAGGATCAAGTGAAGTGCCTG-3′ and T4102D Reverse 5′-CAGGCACTTCACTTGATCCTCTTCTGAAAGCCCAC-3′.

### Immunoblotting

Immunoblotting was performed as previously described (24). The following antibodies were used in this study: anti-DNA-PK_cs_ phospho-T4102 (made in this study as described above), anti-DNA-PK_cs_ phospho-S2056 (Abcam, ab124918), anti-ATM phospho-S1981 (Abcam, ab81292), anti-CHK2 phospho-T68 (Cell Signaling, 2197), anti-LIG4 (Cell Signaling, 14649), anti-Ku80 (Santa Cruz, sc-17789), anti-Ku70 (Santa Cruz, sc-515736), anti-XRCC4 (Santa Cruz, sc-271087), anti-XLF (Santa Cruz, sc-166488), anti-GFP (Santa Cruz, sc-8334), anti-KAP1 (Bethyl Laboratories A300-274A), anti-KAP1 phospho-S824 (Bethyl Laboratories, A300-767A), anti-tubulin (Sigma-Aldrich, T5168), anti-FLAG M2 (Sigma-Aldrich, F1804), anti-phospho-H2AX (S139) (EMD Millipore, 05-636), and anti-Histone H3 antibody (Biolegend, 819411). Mouse monoclonal antibodies against DNA-PK_cs_ (Clone # 25-4) were produced in house. Secondary antibodies used include anti-mouse IgG (HRP-linked) (Cell Signaling, 7076) and anti-rabbit IgG (HRP-linked) (Cell Signaling, 7074). Image J (Version 1.53e) was used to quantify the intensities of protein bands in the immunoblots.

### Immunoprecipitation (IP) assays

To detect phosphorylation of DNA-PK_cs_ at T4102, V3 cells stably expressing YFP-tagged wild-type DNA-PK_cs_ were irradiated with a dose of 10 Gy and allowed to recover for 30 min. The cells were then washed twice with cold PBS, harvested, and lysed using IP lysis buffer (50 mM Tris-HCl pH 7.4, 500 mM NaCl, 0.5% NP-40, and 10% glycerol with 1× protease and phosphatase inhibitor cocktails (Thermo Fisher)). The lysates were sonicated on ice and then cleared of cellular debris by centrifuging at 20 000 × g for 30 min. 2 mg of total protein was incubated with the DNA-PK_cs_ monoclonal antibody (25-4) and Protein A/G beads (Thermo Fisher) overnight at 4°C with mixing. The following day the beads were washed three times with lysis buffer and then twice with washing buffer (50 mM Tris-HCl pH 7.4, 150 mM NaCl, 0.5% NP-40 and 10% glycerol). Following the final wash, the beads were divided two parts, with one resuspended in 1× SDS sample buffer and other treated with lambda protein phosphatase (λPP) (New England Biosciences) at 30°C for 30 min to remove phosphorylation events, the samples were washed, and finally resuspended in 1× SDS sample buffer. The samples were resolved via SDS-PAGE and phosphorylation at T4102 was assessed via immunoblotting.

To investigate protein-protein interactions, CHO V3 cells and V3 cells complemented with WT, T4102A, or T4102D were irradiated with a dose of 10 Gy and allowed to recover for 10 min. Subsequently, the irradiated cells were washed three times in cold PBS, harvested, and lysed using IP Lysis buffer (50 mM Tris-HCl pH 7.4, 150 mM NaCl, 2 mM MgCl_2_, 0.4% NP-40, 0.6% Triton X-100, 1× protease inhibitor cocktail, 1× phosphatase inhibitor cocktail 2, 20 U/ml Benzonase (Novagen)) on ice. The lysates were sonicated on ice and then cleared of cellular debris by centrifuging at 20 000 × g for 30 min. 2 mg of total protein was incubated with 2 μg anti-GFP antibody, and 30 μl of Protein A/G magnetic agarose beads (Thermo Fisher) overnight with spinning at 4°C. The beads were washed with IP washing buffer (20 mM Tris-HCl pH 7.4, 150 mM NaCl, 0.2% Triton X-100) for 5 times, and boiled in 1× SDS sample buffer. The samples were resolved via SDS-PAGE and immunoblotting was performed for the proteins indicated in the figures.

### *In vitro* phosphorylation assay

Purification of ATM from HT1080 cells stably expressing FLAG-tagged ATM and DNA-PK_cs_ from CHO V3 cells stably expressing YFP-tagged DNA-PK_cs_ was performed as previously described with some modifications (23). To activate the ATM protein, the cells were irradiated with a dose of 10 Gy and allowed to recover for 30 min. Next, the cells were washed three times with cold PBS, harvested, and lysed using Purification Lysis Buffer (50 mM Tris-HCl pH 7.4, 500 mM NaCl, 0.5% NP-40, and 10% glycerol with 1× protease and phosphatase inhibitor cocktails (Thermo Fisher)). The lysates were sonicated on ice and then cleared of cellular debris by centrifuging at 20 000 × g for 30 min. 2 mg of total proteins was incubated with 30 μl M2 FLAG magnetic beads (Sigma-Aldrich) for ATM and 2 μg DNA-PK_cs_ antibody (25-4) and 30 μl Protein A/G beads for DNA-PK_cs_. After an overnight incubation at 4°C, the beads were washed twice with Purification Lysis Buffer, twice with Wash Buffer 1 (50 mM Tris-HCl pH 7.4, 150 mM NaCl, 0.5% NP-40 and 10% Glycerol) and finally twice with Wash Buffer 2 (20 mM Tris-HCl pH 7.4, 50 mM KCl, and 10% Glycerol). For ATM protein, half of beads were resuspended in 1× SDS sample buffer and the sample was resolved via SDS-PAGE, and the other half was for the *in vitro* phosphorylation assay. To remove phosphorylations associated with the purified DNA-PK_cs_ protein, the beads were further equilibrated in 1× λPP reaction buffer (New England Biosciences) and treated with λPP at room temperature for 30 min. The sample was washed using the protocol described above. One-third of the beads containing DNA-PK_cs_ was resolved via SDS-PAGE, and the rest were utilized for the ATM-mediated *in vitro* phosphorylation assay. The SDS-PAGE gel that resolved purified YFP-DNA-PK_cs_ and FLAG-ATM was Coomaissie Blue stained to show purification of each protein.

*In vitro* phosphorylation of DNA-PK_cs_ by ATM was conducted by mixing beads containing YFP-DNA-PK_cs_ with beads containing FLAG-tagged ATM or control beads. The bead mixtures were placed in Kinase Reaction Buffer (20 mM HEPES 7.4, 50 mM KCl, 2 mM MgCl_2_, 2 mM ATP, 1 mM DTT and 5% glycerol) and incubated at 37°C for 1 h with mixing. The reactions were terminated by adding 1× SDS sample buffer and boiling the samples at 95°C for 5 min. The phosphorylation of DNA-PK_cs_ at T4102 was then assessed by immunoblotting with the pT4102 antibody.

### *In vitro* kinase assay

DNA-PK_cs_ kinase activity was assessed by examining phosphorylation of H2AX peptide *in vitro*, using a modified version of our published protocol (24). Briefly, the reaction was conducted in a 10 μl mixture containing 1× kinase buffer (25 mM Tris-HCl, pH 7.9, 5 mM MgCl_2_, 1 mM DTT, 25 mM KCl and 10% glycerol), 0.1 μg sonicated herring DNA, 20 nM Ku70/80, 0.1 μg biotin-labeled H2AX (biotin-AVGKKASQASQEY) and 2.5 μg of nuclear extract from V3, V3 + DNA-PK_cs_ WT, V3 + DNA-PK_cs_ T4102A, or DNA-PK_cs_ T4102D cell lines. After a 30 min incubation at 30°C, the reactions were terminated by the addition of 1 μl 0.5 M EDTA, and the biotinylated-H2AX peptide was captured using a SAM2 Biotin Capture Membrane (Promega) and the membrane was washed following the manufacturer's suggested protocol. H2AX phosphorylation was detected using an anti-H2AX pS139 antibody via immunoblotting and signal was quantified using ImageJ (1.53e). Background phosphorylation of H2AX was observed in the DNA-PK_cs_ null V3 cell line and this was subtracted from the other samples’ readouts. 100% kinase activity was normalized using the V3 cells expressing WT human DNA-PK_cs_. The reported results are derived from three independent experiments.

### Fluorescent immunostaining and microscopy

IR-induced 53BP1 foci kinetics were monitored in G1 cells as previously described with modifications (25,26). Briefly, CHO V3 cells and V3 cells complemented with WT, T4102A or T4102D were seeded on ‘PTFE’ Printed Slides (Electron Microscopy Sciences) and two days later the cells were mock treated or irradiate with a dose of 2 Gy. At different time points after IR (0.5, 1, 3 or 7 h), the cells were washed twice with cold PBS and fixed with 4% paraformaldehyde (in PBS) for 20 min at room temperature, washed five times with PBS, and incubated in 0.5% Triton X-100 on ice for 10 min. Cells were washed five times with 1 × PBS and incubated in blocking solution (5% goat serum (Jackson Immuno Research) in 1 × PBS) for 1h. The blocking solution was replaced with the 53BP1 (ab175933, Abcam) and Cyclin A2 (ab16726, Abcam) primary antibodies (1:1000 dilution for both antibodies) diluted in 5% normal goat serum in 1 × PBS and the cells were incubated at 4°C overnight. The next day the cells were washed five times with Wash Buffer (1% BSA in 1 × PBS). Next, the cells were incubated with anti-rabbit IgG conjugated with Alexa Fluor 488 (Molecular Probes) and anti-mouse IgG conjugated with Texas Red (Molecular Probes) (1:1000 dilution for both antibodies) secondary antibodies in 1% BSA, 2.5% goat serum in 1 × PBS for 1 h in the dark, followed by five washes. After the last wash, the cells were mounted in VectaShield Antifade mounting medium containing 4′,6-diamidino-2-phenylindole (DAPI). Images were acquired using a Zeiss AxioImager fluorescence microscope utilizing a 63 × oil objective lens. The 53BP1 foci were only counted in the cells with no Cyclin A staining.

### Laser micro-irradiation and real-time recruitment

Real-time recruitment of fluorescent tagged DNA-PK_cs_ WT, T4102A, T4102D, Ku80, XRCC4, XLF and PNKP in response to DSB induction was examined following laser micro-irradiation with a Carl Zeiss Axiovert 200M microscope with a Plan-Apochromat 63×/NA 1.40 oil immersion objective (Carl Zeiss) as previously described (24,26). The following cell lines were used in this study to measure recruitment of the proteins to laser-generated DSBs. For DNA-PK_cs_, CHO V3 cells stably expressing YFP-tagged DNA-PK_cs_ WT, T4102A, or T4102D. For Ku80, XRCC4, XLF and PNKP recruitment, GFP-tagged Ku80, XRCC4, XLF or PNKP was transiently expressed in CHO V3 cells complemented with FLAG-tagged DNA-PK_cs_ WT, T4102A or T4102D. The YFP/GFP or FLAG tag was attached the N-terminus in all proteins. The cells were seeded on a 35 mm glass-bottomed dish (Mattek) and incubated with 10 μM BrdU. 24 h later, the medium was replaced with CO_2_-independent medium and placed in a chamber on the microscope that was set at 37°C. To generate laser-induced DSBs, a 365-nm pulsed nitrogen laser (Spectra-Physics, Catalog #VSL337NDS2, purchased in May 2020) was set at 80% of maximum power output and micro-irradiation was performed using the pulsed nitrogen laser. Time-lapse images were taken using an AxioCam HRm camera (Carl Zeiss). Carl Zeiss Axiovision software (v4.91) was used to measure fluorescence intensities of the micro-irradiated and control areas, and the resulting intensity of irradiated area was normalized to non-irradiated control area to obtain the alteration of the interested proteins as described previously (24,26).

### Subcellular fractionation

The accumulation of DNA damage response proteins to chromatin following IR-induced DNA damage was examined as previously described with some modifications (27). Briefly, the V3 cells expressing either DNA-PK_cs_ WT or T4102A were mock-treated or irradiated with 10 Gy, allowed to recover for 10 or 30 min before being harvested. The harvested cells were incubated with CSK100 buffer containing 10 mM PIPES pH 6.8, 100 mM NaCl, 300 mM sucrose, 3 mM MgCl_2_, 1 mM EGTA, 0.2% Triton X-100 and protease inhibitor and phosphatase inhibitor cocktails (Thermo Fisher) for 40 min at 4°C. The cells were then pelleted by centrifugation at 5000 × g and the resulting pellets were lysed with buffer 1 containing 50 mM HEPES pH 7.5, 50 mM NaCl, 0.05% SDS, 2 mM MgCl_2_, 10% glycerol, 0.1% Triton X-100, 1× protease inhibitor and phosphatase inhibitor cocktails, and 10 Units of RNase-free DNase I. The chromatin proteins were included in the supernatant after centrifugation at 18 000 × g. The protein concentration of each sample was measured using a Pierce BCA Protein Assay kit (Thermo Fisher). 20 μg of each fraction was resolved via SDS-PAGE, and then transferred to a PVDF membrane for immunoblotting.

### NHEJ assay

CHO cells were seeded in triplicate at a density of 40 000 cells per well in 12-well plates. The following day, cells were transfected with reporter plasmids using Lipofectamine 3000 (ThermoFisher) according to the manufacturer protocol. For each cell line, one well was transfected with undamaged plasmid cocktail, one well was transfected with NHEJ plasmid cocktail, and one well was left untransfected. After 24 h, cells were trypsinized and analyzed by flow cytometry using an Attune NxT flow cytometer. Compensation and gating were established by running untransfected cells along with cells transfected with individual undamaged plasmids expressing wild type fluorescent proteins (single color controls) as described previously (28). Each experiment was conducted three times on separate days.

Reporter plasmid cocktails were as follows: undamaged plasmid cocktail: 50 ng pMax_BFP, 50 ng pMax_mOrange, 500 ng non-fluorescent carrier plasmid pDDCMV. NHEJ plasmid cocktail: 50 ng BFP_NHEJ, 50 ng pMax_mOrange, 500 ng non-fluorescent carrier plasmid pDDCMV. Reporter fluorescence was used as a measurement of NHEJ efficiency as described in Piett *et al.* and below:


$$\begin{eqnarray*}\% \ Reporter\ Expression\ = \frac{X}{Y}\ \end{eqnarray*}$$

$$\begin{eqnarray*}&& X\ \left( {NHEJ\ plasmid\ cocktail} \right)\ \nonumber\\ &=& \frac{{BFP\_NHEJ\ Count \times Mean\ BFP\_NHEJ\ Intensity}}{{mOrange\ Count \times Mean\ mOrange\ Intensity}}\ \end{eqnarray*}$$

$$\begin{eqnarray*} && Y\ \left( {undamaged\ cocktail} \right)\ \nonumber\\ &=& \frac{{\ BFP\ Count \times Mean\ BFP\ Intensity}}{{mOrange\ Count \times Mean\ mOrange\ Intensity}}\ \end{eqnarray*}$$

### Colony formation assay

Cell survival curves were obtained by measuring the colony-forming abilities of irradiated cell populations as previously described (29). CHO V3 cells and V3 cells complemented with YFP-tagged DNA-PK_cs_ WT, T4102A or T4102D were mock treated or irradiated at doses of 1, 2, 4 or 6 Gy, or incubated with different concentration of etoposide or Camptothecin as indicated in the figure legend, and then plated on 60-mm plastic Petri dishes. After 8 days, cells were fixed with 100% ethanol and stained with 0.1% crystal violet in a 100% ethanol solution. Colonies were scored and the mean value for triplicate culture dishes was determined. Cell survival was normalized to plating efficiency of untreated controls for each cell type.

### Chromosome aberration assay

To investigate genome stability following irradiation in CHO V3 cells and V3 cells complemented with WT, T4102A or T4102D, the cells were irradiated with a dose of 2 Gy and then allowed to recover in normal cell culture conditions for 24 hrs. Next, the cells were incubated with Colcemid at concentration of 0.1 μg/ml for 4 h and then harvested by trypsinzation. After washing with PBS at room temperature, the cells were then incubated with warmed (37°C) 75 mM KCl. Samples were processed and chromosomal abnormalities were scored as previously described (25).

## RESULTS

### ATM phosphorylates DNA-PK_cs_ in its FATC domain at threonine 4102 in response to DNA double-stranded breaks

**Figure 1.**
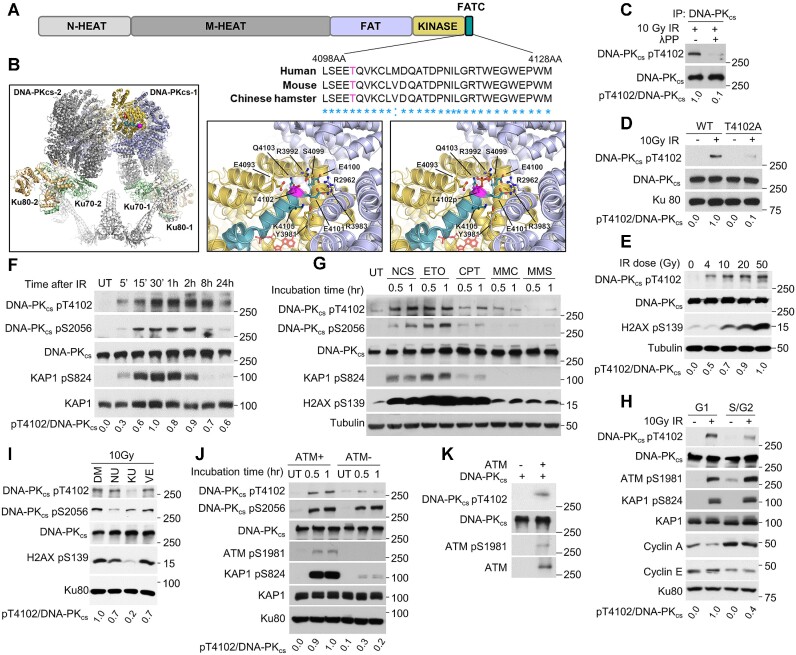
ATM phosphorylates DNA-PK_cs_ in its FATC domain at threonine 4102 in response to DSBs. (**A**) Threonine 4102 (T4102) is conserved in the FATC domain of DNA-PK_cs_ in human, mouse, and Chinese hamster. (**B**) Structure of DNA-PKcs, generated in PyMol version 2.5 from PDB: 7LT3 (overview) and 7OTP (zoomed view) (7,35). T4102 residue is highlighted in magenta. DNA-PK_cs_ domains are highlighted thusly: FATC domain (green), kinase domain (yellow), FAT domain (blue) and N-terminal HEAT repeat domains (gray). Ku70 (light green) and Ku80 (light orange) are highlighted and other NHEJ proteins are in white. Zoomed view compares T4102 with modeled phosphorylated T4102, with nearby polar and charged residues shown as sticks, (**C**) DNA-PK_cs_ is phosphorylated at T4102 (pT4102) after ionizing radiation (IR) and this signal is lost when treated with lambda protein phosphatase (λPP). YFP-tagged DNA-PK_cs_ was immunoprecipitated from CHO V3 cells stably expressing YFP-DNA-PK_cs_ using an anti-DNA-PK_cs_ antibody (25-4) 20 min following treatment with 10 Gy of γ-rays. Half of the beads containing DNA-PK_cs_ were treated with λPP to dephosphorylate the protein. (**D**) Ablating the T4102 phosphorylation site via alanine substitution (T4102A) results in loss of IR-induced phosphorylation at T4102. CHO V3 cells expressing YFP-tagged DNA-PKcs WT or T4102 were irradiated with a dose of 10 Gy of γ-rays, allowed to recover for 20 min, and immunoblotting was performed to examine the phosphorylation of DNA-PK_cs_ at T4102. (**E**) Tracking IR-induced dose-dependent pT4102. CHO V3 cells stably expressing YFP-DNA-PK_cs_ were treated with the indicated doses of IR, allowed to recover for 20 min, and then phosphorylation of DNA-PKcs at T4102 and H2AX at S139 were assessed via immunoblotting. (**F**) Time course of pT4102 following treatment with 10 Gy of γ-rays. CHO V3 cells expressing YFP-tagged DNA-PKcs were treated with a dose of 10 Gy of IR and allowed to recover for the times indicated in the figure. Phosphorylation of DNA-PK_cs_ at S2056 and T4102, and KAP1 at S824 were assessed via immunoblotting. (**G**) Phosphorylation of DNA-PK_cs_ at T4102 following treatment with various DNA damaging agents. CHO V3 cells stably expressing YFP-DNA-PKcs were treated for 1 h with 200 ng/ml neocarzinostatin (NCS), 1 μM etoposide (ETO), 1 μM camptothecin (CPT), 0.5 μg/ml mitomycin C (MMC) or 50 μg/ml methyl methanesulfonate (MMS). Cells were then harvested and immunoblotting was performed to assess phosphorylation of DNA-PK_cs_ at S2056 and T4102, KAP1 at S824, and H2AX at S139. (**H**) Cell cycle-dependent phosphorylation of DNA-PK_cs_ at T4102 following IR. Cells were treated with a double thymidine blocked and then released and collected at selected times to enrich cells in G1 and S/G2 phases of the cell cycle, irradiated with a dose of 10 Gy, and harvested 30 min later. Phosphorylation of DNA-PK_cs_ at T4102, KAP1 at S824, and ATM at S1981 were assessed via immunoblotting. (**I**) Inhibition of ATM, but not ATR or DNA-PK_cs_, blocks IR-induced DNA-PK_cs_ phosphorylation at T4102. CHO V3 cells stably expressing YFP-DNA-PK_cs_ were pretreated with DMSO, 5 μM DNA-PKcs inhibitor NU7441 (NU), ATM inhibitor KU55933 (KU) or ATR inhibitor VE821 (VE) for 2 h and then irradiated with a dose of 10 Gy and allowed to recover for 30 min. Cells were then harvested and immunoblotting was performed to assess phosphorylation of DNA-PK_cs_ at S2056 and T4102 and H2AX at S139. (**J**) IR-induced phosphorylation of DNA-PK_cs_ at T4102 is lost in the ATM-deficient cell line AT5BIVA. ATM-deficient AT5BIVA cells and the cells stably expressing ATM were irradiated with a dose of 10Gy of γ-rays and allowed to recover for 0.5 or 1 h. Cells were then harvested and immunoblotting was performed to assess phosphorylation of DNA-PK_cs_ at S2056 and T4102, KAP1 at S824, and ATM at S1981. (**K**) ATM phosphorylates DNA-PK_cs_*in vitro*. FLAG-tagged ATM was isolated from irradiated HT1080 cells stably expressing FLAG-tagged ATM using anti-FLAG M2 sepharose. DNA-PKcs was isolated from untreated CHO V3 cells stably expressing YFP-tagged DNA-PKcs using an anti-DNA-PKcs antibody (25-4). The two samples were mixed together in a kinase reaction buffer for 1 hr. The reaction was terminated and immunoblotting was performed to assess phosphorylation of DNA-PKcs at T4102 and ATM at S1981.

DNA-PK_cs_ is composed of HEAT (Huntington-elongation factor 3A- PP2A subunit-TOR) repeats, which are differentiated into a unique N-terminal domain (N-HEAT, amino acids 1-892) and a central unit called the M-HEAT domain (amino acids 893-2801) and a C-terminal region that contains the kinase domain (amino acids 3565-4100), which is flanked N-terminally by the FAT (FRAP, ATM, TRRAP) domain and C-terminally by the FATC (FAT C-terminal) domain (30). The FATC domain comprises the extreme C-terminus of DNA-PK_cs_ and is a highly conserved domain of approximately 30 amino acids (31). Early work showed that the FATC domain is indispensable for DNA-PK_cs_ activity, as deletion or mutagenesis in this domain deleteriously affects the function of DNA-PK_cs_ (32). This drove us to postulate that modulation of DNA-PK_cs_ may be regulated by a post-translational modification in the FATC domain. Mass spectrometry analysis of an *in vitro* auto phosphorylation assay previously identified that DNA-PK_cs_ is phosphorylated in the FATC domain at threonine T4102 (pT4102) (33). However the biological relevance of this phosphorylation event has not been previously investigated, propelling us to determine if this site regulates the function of DNA-PK_cs_. Alignment of DNA-PK_cs_ orthologues identified that T4102 is highly conserved in most primates, rodents, and birds (Figure 1A and Supplementary Figure S1), but this site is less conserved in other vertebrates (Supplementary Table S1) (34). Analysis of the DNA-PK_cs_ structure shows that T4102 is surface exposed and thus a viable phosphorylation target (Figure 1B) (7,35). T4102 is positioned at the beginning of the DNA-PK_cs_ FATC domain and proximal to the FAT and kinase domains, and in the context of the NHEJ long-range synaptic complex it is not near the DNA-PK_cs_ dimer interface, DNA or other core NHEJ proteins. A model of phosphorylated T4102 shows that the phosphoryl group will clash with S4099 and repel E4093 within the kinase domain (Figure 1B). Conformational changes within DNA-PK_cs_ will therefore be required to accommodate phosphorylated T4102, which may be facilitated by several other charged or polar residues from the FAT, kinase, and FATC sub-domains in this region.

To examine if DNA-PK_cs_ is phosphorylated at this amino acid after DNA damage in cells, a phospho-specific antibody to T4102 was generated and validated using dot blot analysis (Supplementary Figure S2A). DNA-PK_cs_ is phosphorylated at T4102 (pT4012) following exposure to ionizing radiation (IR) and the signal was lost when the sample was treated with lambda phosphatase, supporting that the antibody recognizes a phosphorylation event (Figure 1C). Next, the specificity of the phospho-antibody was assessed by complementing the DNA-PK_cs_ deficient Chinese Hamster Ovary (CHO) cell line V3 with YFP-tagged wild-type DNA-PK_cs_ (WT) or DNA-PK_cs_ in which the phosphorylation site at T4102 was ablated via alanine substitution (T4102A) (Supplementary Figure S2B). The signal was significantly decreased in cells expressing the T4102A mutant protein, indicating the antibody specifically recognizes pT4102 (Figure 1D). Furthermore, pT4102 occurs following IR exposure in a dosage-dependent manner (Figure 1E), with observation of the pT4102 signal starting at 5 minutes, peaking at 30–60 min, and still detectable 24 h post-IR (Figure 1F). This phenotype is conserved in human cells, as pT4102 initiates 5 min and peaks 60 min post-IR in the human cell line U2OS (Supplementary Figure S2C), and IR-induced pT4102 is also observed in HeLa cells (Supplementary Figure S2D). In addition to IR, treatment with the radiomimetic agent neocarzinostatin (NCS) and the topoisomerase 2 inhibitor etoposide (ETO) induced robust pT4102 signal (Figure 1G). Modest phosphorylation of DNA-PK_cs_ at T4102 was observed after treatment with the topoisomerase 1 inhibitor camptothecin (CPT), but limited to no phosphorylation occurred following treatment with the DNA cross-linking agent mitomycin (MMC) and DNA alkylating agent methyl methanesulfonate (MMS) (Figure 1G). As IR, NCS and ETO directly generate DSBs and CPT-induced DNA damage can be processed to form DSBs, the data suggests that DSBs induce phosphorylation of DNA-PK_cs_ at T4102. We then assessed if IR-induced phosphorylation of DNA-PK_cs_ at T4102 is cell cycle specific. Cells were synchronized via a double thymidine block and then released to allow examination of IR-induced phosphorylation of DNA-PK_cs_ at T4102 in G1 and S/G2 phases of the cell cycle. We observed that pT4102 occurs in both G1 and S/G2 phases, but phosphorylation is more prominent in G1 phase of the cell cycle (Figure 1H). We next aimed to identify the kinase responsible for phosphorylating DNA-PK_cs_ at T4102 in response to DNA damage. As phosphorylation at this site is induced by DSBs, we focused on the DNA damage-responsive kinases, including DNA-PK_cs_, ATM, and ATR. Pretreatment of cells with the inhibitors NU7441, KU55933 and VE-821 to block DNA-PK_cs_, ATM and ATR activity, respectively, shows that pT4102 is significantly attenuated in cells pretreated with KU55933 but not NU7441 or VE-821 (Figure 1I). This data indicates that ATM phosphorylates DNA-PK_cs_ at T4102 in response to DNA damage. To verify this result, we assessed pT4102 in the ATM deficient cell line AT5 and AT5 cells complemented with ATM (36). IR-induced phosphorylation of DNA-PK_cs_ at T4102 is almost completely lost in the ATM-deficient cell line and this was rescued by complementing AT5 cells with wild-type ATM (Figure 1J). Finally, we examined the ability of ATM to phosphorylate DNA-PK_cs_ at T4102 *in vitro*. DNA-PK_cs_ was isolated from cells and incubated with purified ATM in the presence of ATP (Supplementary Figure S3). ATM autophosphorylates at S1981 in this system and it phosphorylates DNA-PK_cs_ at threonine 4102 as monitored by the pT4102 antibody (Figure 1K). Collectively, the data illustrates that ATM phosphorylates DNA-PK_cs_ at T4012 in response to DNA damage.

### Ablating the T4102 phosphorylation site attenuates DNA-PK_cs_ activity and destabilizes the interaction between DNA-PK_cs_ and the Ku-DNA complex

**Figure 2.**
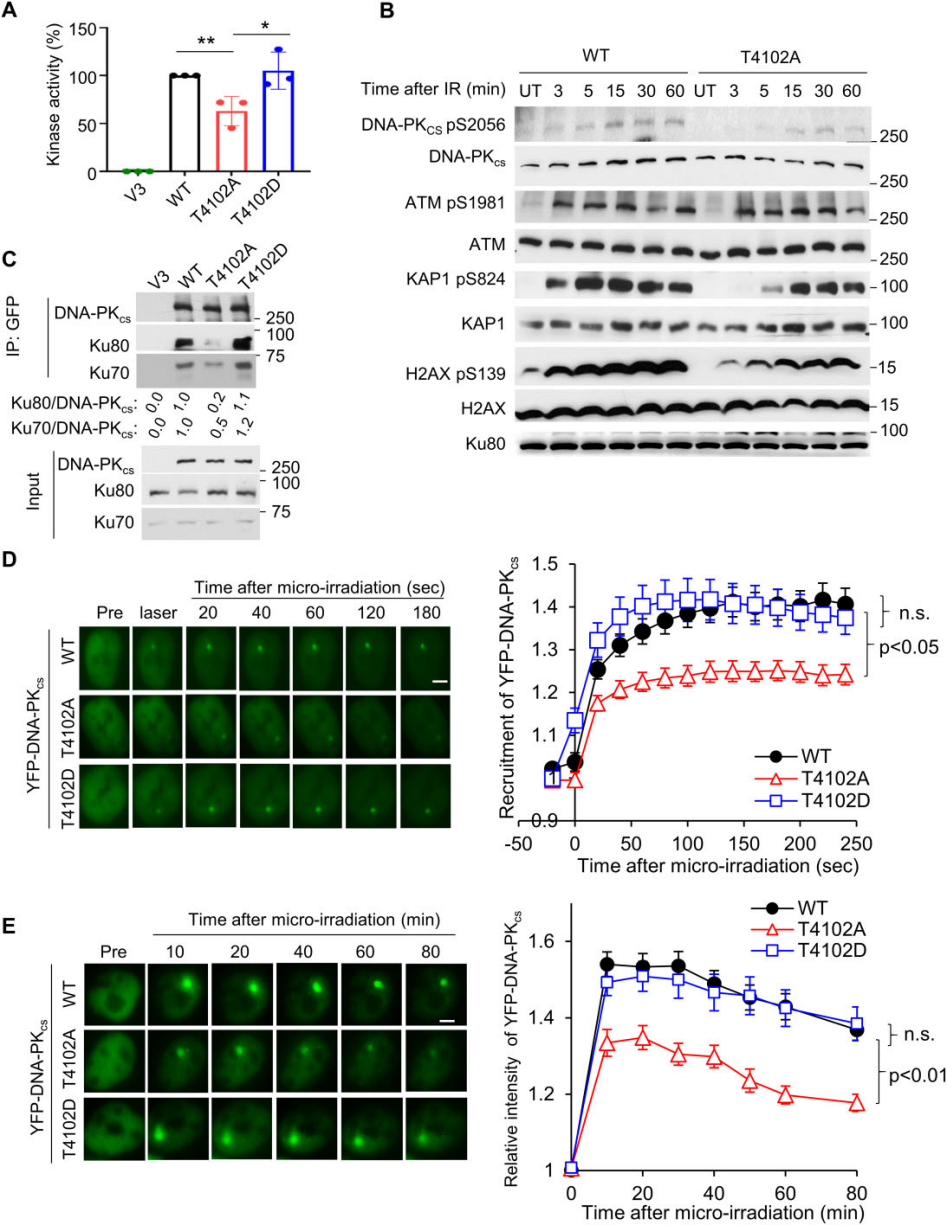
Ablating the T4102 phosphorylation site attenuates DNA-PK_cs_ activity and destabilizes the interaction between DNA-PK_cs_ and the Ku-DNA complex. (**A**) Measurement of DNA-PK_cs_*in vitro* kinase activity. Nuclear extracts from the CHO V3 cells and those stably expressing DNA-PK_cs_ WT, T4102A, and T4102D were examined for their ability to phosphorylate a biotin-tagged H2AX peptide. H2AX phosphorylation was observed in the CHO V3 cells (DNA-PK_cs_ null) cell line and this was subtracted from the other samples’ readouts. The 100% kinase activity was normalized using the DNA-PK_cs_ WT cell lysate results. The data are presented as the mean ± SD from three individual experiments. (**B**) Blocking DNA-PK_cs_ phosphorylation at T4102 suppresses IR-induced DNA-PK_cs_-mediated phosphorylation events. CHO V3 cells stably expressing YFP-tagged DNA-PKcs WT and T4102A were irradiated using a dose of 10 Gy of γ-rays and then allowed to recover at times indicated in the figure. Phosphorylation of DNA-PKcs at S2056, ATM at S1981, KAP1 at S824, and H2AX at S139 were assessed via immunoblotting. (**C**)T4102 phosphorylation promotes stabilization of the interaction between DNA-PK_cs_ and the Ku70/80 heterodimer after IR. CHO V3 cells and V3 cells stably expressing YFP-tagged DNA-PK_cs_ WT, T4102A and T4102D were treated with a dose of 10 Gy of IR and allowed to recover for 10 min and whole cell lysates were generated. DNA-PK_cs_ was pulled down from the lysates using ChromoTek GFP-Trap® Agarose beads and immunoblotting was performed to verify immunoprecipitation of DNA-PK_cs_ and the ability of Ku70 and Ku80 to interact with DNA-PKcs was examined. (**D**) Ablating T4102 phosphorylation attenuates the initial recruitment (up to 5 min) of DNA-PK_cs_ to laser-induced DSBs. Relative fluorescent intensity of YFP-tagged wild type DNA-PK_cs_ WT, T4102A, and T4102D are presented as mean ± SEM and significance was assessed via a student's t-test. The bar indicates 5 μm. (**E**) T4102 phosphorylation promotes accumulation/retention (up to 80 min) of DNA-PK_cs_ at laser-induced DSBs. Relative fluorescent intensity of YFP-tagged wild type DNA-PK_cs_ WT, T4102A and T4102D are presented as mean ± SEM and significance was assessed via a student's t-test. The bar indicates 5 μm.

As T4102 lies in the FATC domain, we next assessed if phosphorylation of this site modulates DNA-PK_cs_ kinase activity. We complemented V3 cells with YFP-tagged WT, T4102A, and the DNA-PK_cs_ mutation in which T4102 was mutated to aspartic acid to mimic phosphorylation at this amino acid (T4102D) (Supplementary Figure 2B). Subsequently, we examined the kinase activities of these proteins *in vitro* using H2AX peptide as a substrate (24). We observed that the T4102A mutant protein had significantly reduced (∼ 40% decrease) activity compared to WT and T4102D proteins *in vitro* (Figure 2A). Next, we assessed DNA-PK_cs_-mediated phosphorylation in response to DSBs *in vivo*. Previously, we and others identified that at early time points (3-15 minutes) post-IR treatment that DNA-PK_cs_ phosphorylates H2AX at S139 and KAP1 at S824 (24,37). Therefore, we examined DNA-PK_cs_ autophosphorylation and phosphorylation of H2AX and KAP1 in V3 cells complemented with DNA-PK_cs_ WT and the kinase defective T4102A in response to IR-generated DSBs at these times points. We found that IR-induced autophosphorylation of DNA-PK_cs_ at S2056 is decreased in T4102A cells compared to WT cells (Figure 2B). Furthermore, phosphorylation of H2AX and KAP1 are decreased in T4102A cells at early time points post-IR treatment (Figure 2B). This data illustrates that ablating the T4102 phosphorylation sites attenuates DNA-PK_cs_ activity *in vitro* and *in vivo*. Next, we determined if the decrease in DNA-PK_cs_-mediated phosphorylation events was due to a decrease in the DNA-PK_cs_-Ku interaction. We found that T4102A has decreased interaction with Ku following exposure to IR, compared to WT and T4102D (Figure 2C). Consistent with previous results, no strong interaction between DNA-PK_cs_ WT, T4102A, or T4102D and Ku was detected in the absence of DNA damage (Supplementary Figure S4A) (38–40). The disruption of the DNA-PK_cs_-Ku interaction was further examined via monitoring the dynamics of DNA-PK_cs_ at laser-generated DSBs. We found that blocking T4102 phosphorylation results in decreased recruitment of DNA-PK_cs_ to DSBs compared to WT and the T4102D proteins (Figure 2D). Moreover, kinetic analysis shows that the T4102A mutant prematurely dissociates from laser-generated DSBs compared to the WT and T4102D proteins (Figure 2E). As ATM mediates the phosphorylation of DNA-PK_cs_ at T4102, we examined if blocking ATM kinase activity affects the dynamics of DNA-PK_cs_ at laser-generated DSBs. We found that inhibition of ATM kinase activity using the small molecule KU55933 did not alter the initial recruitment of DNA-PK_cs_ to laser-induced DSBs (10 s after DSB induction), but significantly reduced accumulation/retention of DNA-PK_cs_ at the DNA damage site (Supplementary Figure S4B). Collectively, the data illustrates that blocking phosphorylation of DNA-PK_cs_ at T4102 results in a decrease in DNA-PK_cs_ kinase activity, resulting in destabilization the DNA-PK_cs_-Ku complex.

### Stabilization of the NHEJ machinery at DSBs is promoted by phosphorylation of DNA-PK_cs_ at T4102

**Figure 3.**
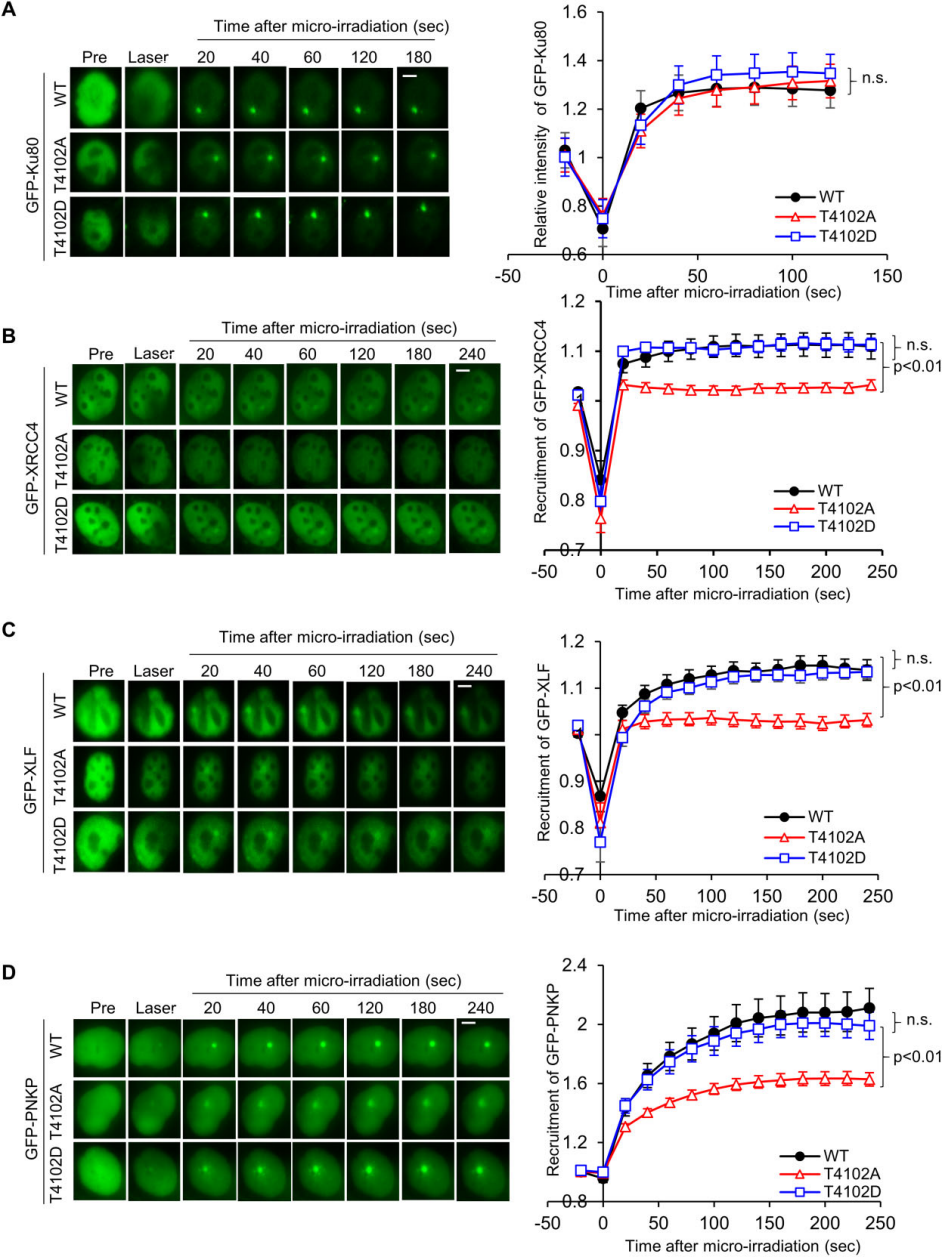
Stabilization of the NHEJ machinery at DSBs is promoted by phosphorylation of DNA-PK_cs_ at T4102. (**A**) Phosphorylation of DNA-PK_cs_ at T4102 is not required for the recruitment of Ku80 to laser-induced DSBs. Recruitment of GFP-Ku80 was monitored in CHO V3 cells stably expressing FLAG-tagged DNA-PK_cs_ WT, T4102A and T4102D. The data are presented as mean ± SEM with *P*-values calculated via Student's *t*-test. n.s., not significant. (B-D) Ablating T4102 phosphorylation attenuates the recruitment of GFP-XRCC4 (**B**), GFP-XLF (**C**) and GFP-PNKP (**D**) to laser-generated DSBs. Relative fluorescent intensity of GFP-tagged XRCC4 (B), XLF (C) and PNKP (D) in CHO V3 cells with FLAG-tagged DNA-PKcs WT, T4102A and T4102D are presented as mean ± SEM and significance was assessed via a Student's *t*-test. The bar indicates 5 μm.

As DNA-PK_cs_ has been implicated in promoting the long-range synaptic complex and T4102 promotes stabilization of the DNA-PK_cs_-Ku complex, we next assessed if phosphorylation at this site affects the dynamics of the NHEJ machinery at DSBs. First, the recruitment of the core NHEJ factors Ku80, XRCC4 and XLF to laser-generated DSBs was examined in V3 cells complemented with FLAG-tagged DNA-PK_cs_ WT, T4102A and T4102D (Supplementary Figure S5A). The initial dynamics of Ku80 is similar in all three cell lines, illustrating that T4102 phosphorylation does not regulate the ability of the Ku heterodimer to bind to DSBs or its initial dynamics at DSBs (Figure 3A). We observed that GFP-tagged XRCC4 and XLF are quickly recruited to laser-induced DSBs in WT, T4102A, and T4102D cells (Figure 3B, C). However, XRCC4 and XLF signal wanes in the T4102A complemented cells compared to the WT and T4102D cells (Figure 3B, C), suggesting that stability of the NHEJ machinery is decreased when phosphorylation of DNA-PK_cs_ at T4102 is blocked. Moreover, we extended this to the DNA end processing factor PNKP. Similar to XRCC4 and XLF, PNKP recruitment/retention at laser-generated DSBs was decreased in T4102A complemented cells compared to WT and T4102D (Figure 3D). Finally, we observed that IR-induced recruitment of XRCC4, LIG4, and DNA-PK_cs_ to the chromatin faction was significantly decreased in the T4102A cells compared to WT cells, supporting the observation that blocking T4102 phosphorylation affects the dynamics of NHEJ factors at DSBs (Supplementary Figure S5B). Together, the data show that ATM-mediated phosphorylation of DNA-PK_cs_ at T4102 promotes stabilization of the NHEJ machinery at DSBs.

### Phosphorylation of DNA-PK_cs_ at T4102 is important for NHEJ-mediated DSB repair

**Figure 4.**
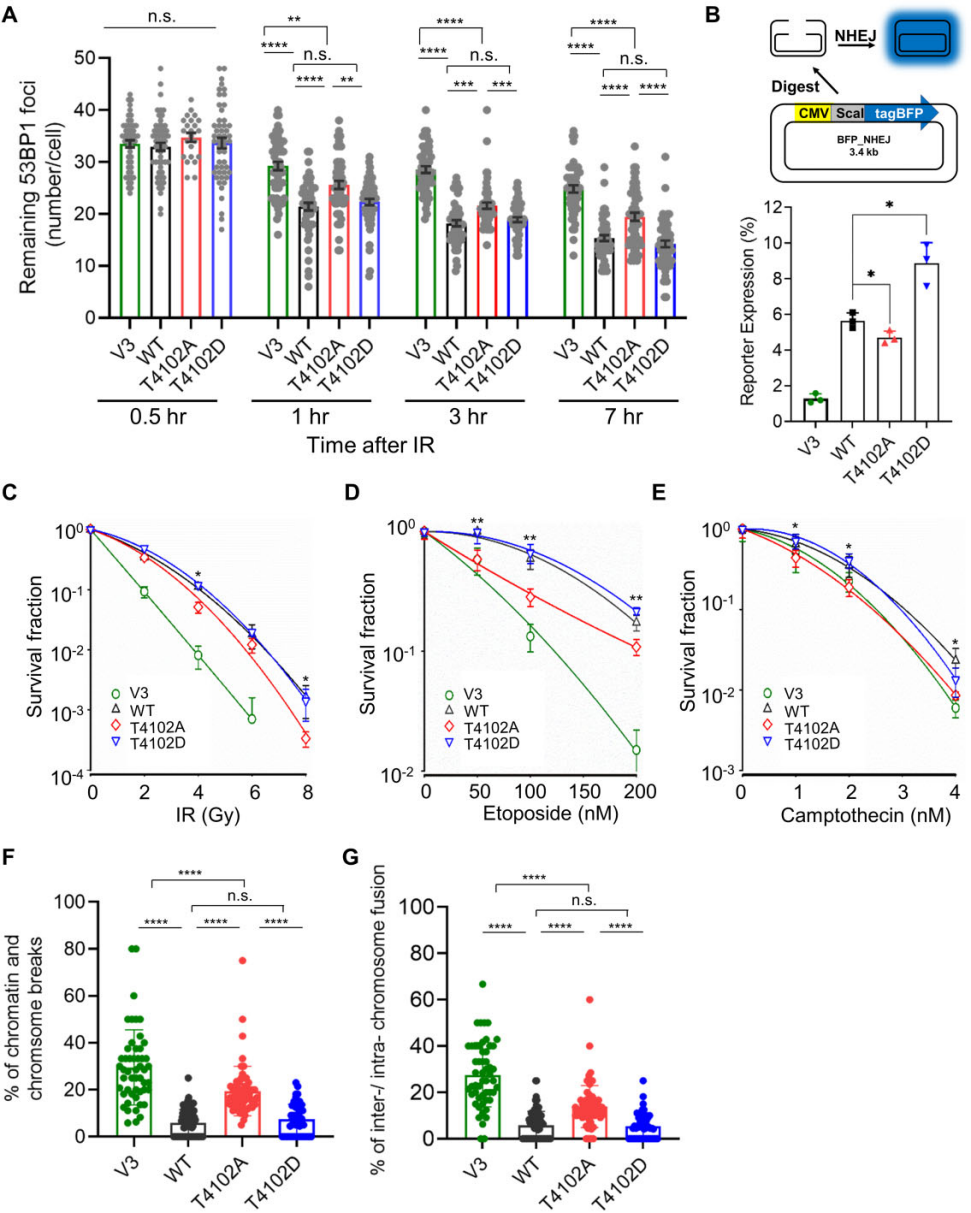
Phosphorylation of DNA-PK_cs_ at T4102 is important for NHEJ-mediated DSB repair. (**A**) IR-induced 53BP1 foci resolution in G1 cells is attenuated in T4102A cells compared to WT and T4102D cells. CHO V3 cells and V3 cells complemented with FLAG-tagged DNA-PK_cs_ WT, T4102A or T4102D were irradiated with 2 Gy of γ-rays and 53BP1 foci formation and resolution was assessed 0.5, 1, 3 and 7 h post-IR. 53BP1 foci at each time point were calculated in over 50 Cyclin A-negative cells and the data are presented as Mean ± SEM. Student's *t*-test (two-sided) was performed to assess statistical significance (n.s., not significant; **P< 0.01; ***P< 0.001; ****P< 0.0001). (**B**) DNA-PK_cs_ phosphorylation at T4102 promotes NHEJ. NHEJ reporter and control plasmids were transfected into CHO V3 cells and V3 cells stably expressing YFP-tagged DNA-PK_cs_ WT, T4102A and T4102D and NHEJ efficiency was calculated using flow cytometry analysis as described in the Materials and Methods. The data are presented as mean ± SD with *P*-value from three independent repeats. *P< 0.05. (C–E) T4102A complemented cells are sensitive to DNA damaging agents including ionizing radiation (**C**), etoposide (**D**) and camptothecin (CPT) (**E**), compared to WT and T4102D cells. Clonogenic survival assays were performed to compare the radiation sensitivities of CHO V3 cells and V3 cells complemented with WT, T4102A or T4102D. Cells were irradiated at the indicated doses or incubated with etoposide or CPT at the indicated concentrations, and plated for analysis of survival and colony-forming ability. Two-sided Student's *t*-test were presented for comparison between V3 cells expressing YFP-tagged DNA-PK_cs_ WT and T4102A. *P< 0.05; **P< 0.01. (F, G) Blocking T4102 phosphorylation increases IR-induced chromosome and chromatin breaks (**F**) and chromosome fusions (**G**). Metaphase spreads were performed following treatment with 2 Gy of IR in CHO V3 cells and V3 cells stably expressing YFP-tagged DNA-PK_cs_ WT, T4102A and T4102D. Chromosome and chromatin breaks as well as inter/intra-chromosome fusions were enumerated in at least 50 cells. Data are presented as mean ± SD and significance was assessed via a two-sided Student's *t*-test. n.s., not significant; ****P< 0.0001.

As blocking phosphorylation of DNA-PK_cs_ at T4102 destabilizes the NHEJ machinery at DSBs, we next examined if blocking phosphorylation at this site affects NHEJ. First, we assessed IR-induced 53BP1 focus formation and resolution in G1 cells, which was used as an indirect marker for DSB repair and NHEJ. To make 53BP1 foci enumerable, we used a dose of 2 Gy of IR, which also induced phosphorylation of DNA-PK_cs_ at T4102 (Supplementary Figure S6A). As shown in Figure 4A, we found at 30 minutes post-IR, the number of 53BP1 foci is the same in V3, WT, T4102A, and T4102D cells. However, at 1-, 3- and 7-hours post-IR, 53BP1 focus resolution was attenuated in V3 and T4102A cells compared to WT and T410D cells. 53BP1 focus resolution is more attenuated in the DNA-PK_cs_-deficient V3 cells compared to the T4102A cells, suggesting that T4102 phosphorylation does not result in a complete abolishment of NHEJ. Next, NHEJ was monitored via a FM-HCR assay (28). We observed that the loss of DNA-PK_cs_ (V3) results in almost complete loss of NHEJ, which was rescued by expression of WT DNA-PK_cs_ (Figure 4B). The T4102A mutant complemented cells have a modest (∼20% decrease), but significant, decrease in NHEJ repair efficiency compared to WT, while the T4102D complemented cells have a significant increase (∼50%) increase in NHEJ efficiency compared to WT cells (Figure 4B), indicating that phosphorylation of T4102 promotes NHEJ. As NHEJ is decreased in T4102A cells, we determined if blocking this phosphorylation event results in increased sensitivity to DNA damaging agents, including radiation, etoposide, and CPT. We observed that T4102A cells are more sensitive to radiation (Figure 4C), etoposide (Figure 4D), and CPT (Figure 4E) than WT and T4102D cells. Finally, IR-generated chromosomal aberrations, as assessed by chromatin and chromosome breaks and chromosome fusions, are increased in T4102A cells compared to WT and T4102D cells (Figure 4F, G). Collectively, the findings in this study show that ATM phosphorylates the FATC domain of DNA-PK_cs_ at T4102 in response to DSBs. Moreover, phosphorylation at T4102 promotes stabilization of the NHEJ machinery at DSBs, and ultimately, NHEJ and genome stability. This study provides further insight into how ATM functions in the response to DSBs by promoting NHEJ.

## DISCUSSION

DNA-PK_cs_ is heavily phosphorylated in response to DSBs (41). The best characterized DNA-PK_cs_ phosphorylation sites include T2609, S2612, T2620, S2624, T2638 and T2647 (collectively called the T2609 or ABCDE cluster) and S2023, S2029, S2041, S2051 and S2056 (termed the S2056 or PQR cluster) and phosphorylation of both clusters is required for NHEJ and modulation of DSB repair pathway choice. The S2056/PQR and T2609/ABCDE phosphorylation clusters have opposing roles in protecting DSBs, with S2056/PQR protecting the DNA ends and T2609/ABCDE promoting DNA end processing (9). Phosphorylation of the T2609/ABCDE cluster functions by destabilizing the binding of DNA-PK_cs_ to the DNA-Ku complex, triggering its release from DSBs to allow access of DNA ends for processing, including the terminal ligation step of NHEJ (40). The kinase activity of DNA-PK_cs_ is modulated by phosphorylation at T3950 and S56/S72, whereas phosphorylation at T946/S1004 inhibits NHEJ without affecting the enzymatic activity of DNA-PK_cs_ (42). Here, we add to the importance of DNA-PK_cs_ phosphorylation in modulating its functionality, as we show that in response to DSBs, ATM phosphorylates DNA-PK_cs_ in the FATC domain of the protein at T4102. This phosphorylation event promotes stabilization of the DNA-PK_cs_-Ku interaction and the stabilization of the NHEJ machinery at DSBs, both of which stimulate NHEJ.

A body of evidence shows that crosstalk occurs between DNA-PK_cs_ and ATM, and that they may cooperatively initiate DSB repair signaling and regulate DSB repair (20,32). First, combined deficiency of DNA-PK_cs_ and ATM leads to synthetic lethality in mice (43,44). Second, DNA-PK_cs_ and ATM phosphorylate many common targets required for DDR signaling and DSB repair, including H2AX, KAP1 and components of the NHEJ pathway, such as XLF and Artemis (2). Third, DNA-PKcs and ATM phosphorylate each other to modulate specific activities. For example, ATM phosphorylates DNA-PK_cs_ at S3205 and at the T2609/ABCDE cluster in response to DNA damage (42,45). ATM-mediated phosphorylation of the T2609/ABCDE cluster is essential for NHEJ and allows the freeing of DNA ends for processing by factors including the endonuclease Artemis and the dissociation of DNA-PK_cs_ to allow DNA end ligation (46–48). DNA-PK_cs_ also phosphorylates the T2609/ABCDE cluster in *trans* (8,47,49,50), suggesting that phosphorylation of this cluster by ATM or DNA-PK_cs_ may be context dependent. Moreover, ATM phosphorylation of DNA-PK_cs_ overcomes DNA-PK_cs_-Ku inhibition of resection *in vitro* (51). Lastly, DNA-PKcs inhibits ATM activity upon DNA damage via phosphorylation at multiple sites to regulate ATM-mediated signaling (52). This study adds to the interplay between DNA-PK_cs_ and ATM and supports another positive role in ATM promoting NHEJ via phosphorylation of DNA-PK_cs_.

A significant number of single molecule studies have produced data showing the makeup of the NHEJ machinery at DNA ends. In particular, two synaptic complexes, the long-range and short-range complexes, have been identified and these complexes are believed to protect the DSB ends and then position them for ligation, respectively (6,7). Although powerful, these studies provide limited insight into the dynamics of the NHEJ machinery at DSBs or how DNA end processing enzymes are recruited. Here, we provide evidence that phosphorylation of DNA-PK_cs_ in its FATC domain at T4102 promotes stabilization of DNA-PK_cs_ at DSBs, which supports assembly and maintenance of the NHEJ core complex at the DNA damage site. We found that ablating the T4102 site attenuates overall DNA-PK_cs_ kinase activity and that this correlates with decreased interaction with the Ku-DNA complex and stabilization of the NHEJ machinery. We hypothesize that the decrease in the NHEJ core complex stability at the DNA damage site is due to the attenuated DNA-PK_cs_ kinase activity in the T4102A mutant. This is supported by previous data showing that the transition from the NHEJ long-range to the short-range complex and recruitment of factors required for DNA end processing are modulated by DNA-PK_cs_ kinase activity (6–9,30). It is also possible that T4102 phosphorylation of DNA-PK_cs_ induces long-range conformational changes in DNA-PK_cs_ that are important for mediating DNA-PK_cs_-mediated protein-protein interactions with the core NHEJ factors. The structural predictions suggest that conformational changes within DNA-PK_cs_ will be required to accommodate phosphorylated T4102, which may be facilitated by residues from the FAT, kinase, or FATC sub-domains in this region. The modeling does not provide evidence that phosphorylation at T4102 will directly affect the interaction between DNA-PK_cs_ with the Ku heterodimer or another core NHEJ factor, but we hypothesize that this phosphorylation event may induce an allosteric conformational change that allows the stabilization of the long-range complex. Furthermore, ablating the T4102 phosphorylation site attenuates the recruitment of the DNA end processing factor PNKP to DSBs, suggesting that DNA-PK_cs_ activity and/or a conformational change upon DNA-PK_cs_ phosphorylation at T4102 may promote the processing of unligatable DSBs. We postulate that this allows ATM protein to ensure the complete repair of DSBs by stabilizing the NHEJ complexes to favor the processing and correct joining of DNA ends.

## Supplementary Material

gkad505_Supplemental_Files

## Data Availability

All study materials will be made available to other researchers. Please contact Anthony J. Davis (anthony.davis@utsouthwestern.edu) for reagents.

## References

[1] Tubbs A. , Nussenzweig A. Endogenous DNA damage as a source of genomic instability in cancer. Cell. 2017; 168:644–656. 28187286 10.1016/j.cell.2017.01.002 PMC6591730

[2] Blackford A.N. , Jackson S.P. ATM, ATR, and DNA-PK: the trinity at the heart of the DNA damage response. Mol. Cell. 2017; 66:801–817. 28622525 10.1016/j.molcel.2017.05.015

[3] Lovejoy C.A. , Cortez D. Common mechanisms of PIKK regulation. DNA Repair (Amst.). 2009; 8:1004–1008. 19464237 10.1016/j.dnarep.2009.04.006 PMC2725225

[4] Davis A.J. , Chen B.P., Chen D.J. DNA-PK: a dynamic enzyme in a versatile DSB repair pathway. DNA Repair (Amst.). 2014; 17:21–29. 24680878 10.1016/j.dnarep.2014.02.020 PMC4032623

[5] Yano K. , Chen D.J. Live cell imaging of XLF and XRCC4 reveals a novel view of protein assembly in the non-homologous end-joining pathway. Cell Cycle. 2008; 7:1321–1325. 18418068 10.4161/cc.7.10.5898

[6] Graham T.G. , Walter J.C., Loparo J.J. Two-stage synapsis of DNA ends during non-homologous end joining. Mol. Cell. 2016; 61:850–858. 26990988 10.1016/j.molcel.2016.02.010 PMC4799494

[7] Chen S. , Lee L., Naila T., Fishbain S., Wang A., Tomkinson A.E., Lees-Miller S.P., He Y. Structural basis of long-range to short-range synaptic transition in NHEJ. Nature. 2021; 593:294–298. 33854234 10.1038/s41586-021-03458-7 PMC8122075

[8] Liu L. , Chen X., Li J., Wang H., Buehl C.J., Goff N.J., Meek K., Yang W., Gellert M. Autophosphorylation transforms DNA-PK from protecting to processing DNA ends. Mol. Cell. 2022; 82:177–189. 34936881 10.1016/j.molcel.2021.11.025 PMC8916119

[9] Cui X. , Yu Y., Gupta S., Cho Y.M., Lees-Miller S.P., Meek K. Autophosphorylation of DNA-dependent protein kinase regulates DNA end processing and may also alter double-strand break repair pathway choice. Mol. Cell Biol. 2005; 25:10842–10852. 16314509 10.1128/MCB.25.24.10842-10852.2005 PMC1316975

[10] Chen X. , Xu X., Chen Y., Cheung J.C., Wang H., Jiang J., de Val N., Fox T., Gellert M., Yang W. Structure of an activated DNA-PK and its implications for NHEJ. Mol. Cell. 2021; 81:801–810. 33385326 10.1016/j.molcel.2020.12.015 PMC7897279

[11] Zhao B. , Rothenberg E., Ramsden D.A., Lieber M.R. The molecular basis and disease relevance of non-homologous DNA end joining. Nat. Rev. Mol. Cell Biol. 2020; 21:765–781. 33077885 10.1038/s41580-020-00297-8 PMC8063501

[12] Lee J.H. , Paull T.T. Cellular functions of the protein kinase ATM and their relevance to human disease. Nat. Rev. Mol. Cell Biol. 2021; 22:796–814. 34429537 10.1038/s41580-021-00394-2

[13] Cortez D. , Wang Y., Qin J., Elledge S.J. Requirement of ATM-dependent phosphorylation of brca1 in the DNA damage response to double-strand breaks. Science. 1999; 286:1162–1166. 10550055 10.1126/science.286.5442.1162

[14] Wang H. , Shi L.Z., Wong C.C., Han X., Hwang P.Y., Truong L.N., Zhu Q., Shao Z., Chen D.J., Berns M.W. et al. The interaction of CtIP and Nbs1 connects CDK and ATM to regulate HR-mediated double-strand break repair. PLoS Genet. 2013; 9:e1003277. 23468639 10.1371/journal.pgen.1003277 PMC3585124

[15] Ahlskog J.K. , Larsen B.D., Achanta K., Sorensen C.S. ATM/ATR-mediated phosphorylation of PALB2 promotes RAD51 function. EMBO Rep. 2016; 17:671–681. 27113759 10.15252/embr.201541455 PMC5341514

[16] Bakr A. , Oing C., Kocher S., Borgmann K., Dornreiter I., Petersen C., Dikomey E., Mansour W.Y. Involvement of ATM in homologous recombination after end resection and RAD51 nucleofilament formation. Nucleic Acids Res. 2015; 43:3154–3166. 25753674 10.1093/nar/gkv160 PMC4381069

[17] Kijas A.W. , Lim Y.C., Bolderson E., Cerosaletti K., Gatei M., Jakob B., Tobias F., Taucher-Scholz G., Gueven N., Oakley G. et al. ATM-dependent phosphorylation of MRE11 controls extent of resection during homology directed repair by signalling through Exonuclease 1. Nucleic Acids Res. 2015; 43:8352–8367. 26240375 10.1093/nar/gkv754 PMC4787824

[18] Goodarzi A.A. , Noon A.T., Deckbar D., Ziv Y., Shiloh Y., Lobrich M., Jeggo P.A. ATM signaling facilitates repair of DNA double-strand breaks associated with heterochromatin. Mol. Cell. 2008; 31:167–177. 18657500 10.1016/j.molcel.2008.05.017

[19] Alvarez-Quilon A. , Serrano-Benitez A., Lieberman J.A., Quintero C., Sanchez-Gutierrez D., Escudero L.M., Cortes-Ledesma F. ATM specifically mediates repair of double-strand breaks with blocked DNA ends. Nat. Commun. 2014; 5:3347. 24572510 10.1038/ncomms4347 PMC3948078

[20] Martin M. , Terradas M., Tusell L., Genesca A. ATM and DNA-pkcs make a complementary couple in DNA double strand break repair. Mutat. Res. Rev. Mutat. Res. 2012; 751:29–35. 22230547 10.1016/j.mrrev.2011.12.006

[21] Zha S. , Guo C., Boboila C., Oksenych V., Cheng H.L., Zhang Y., Wesemann D.R., Yuen G., Patel H., Goff P.H. et al. ATM damage response and XLF repair factor are functionally redundant in joining DNA breaks. Nature. 2011; 469:250–254. 21160472 10.1038/nature09604 PMC3058373

[22] Caron P. , Choudjaye J., Clouaire T., Bugler B., Daburon V., Aguirrebengoa M., Mangeat T., Iacovoni J.S., Alvarez-Quilon A., Cortes-Ledesma F. et al. Non-redundant functions of ATM and DNA-pkcs in response to DNA double-strand breaks. Cell Rep. 2015; 13:1598–1609. 26586426 10.1016/j.celrep.2015.10.024 PMC4670905

[23] Lu H. , Shamanna R.A., de Freitas J.K., Okur M., Khadka P., Kulikowicz T., Holland P.P., Tian J., Croteau D.L., Davis A.J. et al. Cell cycle-dependent phosphorylation regulates RECQL4 pathway choice and ubiquitination in DNA double-strand break repair. Nat. Commun. 2017; 8:2039. 29229926 10.1038/s41467-017-02146-3 PMC5725494

[24] Lu H. , Saha J., Beckmann P.J., Hendrickson E.A., Davis A.J. DNA-pkcs promotes chromatin decondensation to facilitate initiation of the DNA damage response. Nucleic Acids Res. 2019; 47:9467–9479. 31396623 10.1093/nar/gkz694 PMC6765147

[25] Saha J. , Bae J., Wang S.Y., Lu H., Chappell L.J., Gopal P., Davis A.J. Ablating putative Ku70 phosphorylation sites results in defective DNA damage repair and spontaneous induction of hepatocellular carcinoma. Nucleic Acids Res. 2021; 49:9836–9850. 34428289 10.1093/nar/gkab743 PMC8464062

[26] Lu H. , Guan J., Wang S.Y., Li G.M., Bohr V.A., Davis A.J. DNA-pkcs-dependent phosphorylation of RECQL4 promotes NHEJ by stabilizing the NHEJ machinery at DNA double-strand breaks. Nucleic Acids Res. 2022; 50:5635–5651. 35580045 10.1093/nar/gkac375 PMC9178012

[27] Benureau Y. , Pouvelle C., Dupaigne P., Baconnais S., Moreira Tavares E., Mazon G., Despras E., Le Cam E., Kannouche P.L. Changes in the architecture and abundance of replication intermediates delineate the chronology of DNA damage tolerance pathways at UV-stalled replication forks in human cells. Nucleic Acids Res. 2022; 50:9909–9929. 36107774 10.1093/nar/gkac746 PMC9508826

[28] Piett C.G. , Pecen T.J., Laverty D.J., Nagel Z.D. Large-scale preparation of fluorescence multiplex host cell reactivation (FM-HCR) reporters. Nat. Protoc. 2021; 16:4265–4298. 34363069 10.1038/s41596-021-00577-3 PMC9272811

[29] Davis A.J. , Chi L., So S., Lee K.J., Mori E., Fattah K., Yang J., Chen D.J. BRCA1 modulates the autophosphorylation status of DNA-pkcs in S phase of the cell cycle. Nucleic Acids Res. 2014; 42:11487–11501. 25223785 10.1093/nar/gku824 PMC4191403

[30] Chen S. , Lees-Miller J.P., He Y., Lees-Miller S.P. Structural insights into the role of DNA-PK as a master regulator in NHEJ. Genome Instab Dis. 2021; 2:195–210. 34723130 10.1007/s42764-021-00047-w PMC8549938

[31] Lempiainen H. , Halazonetis T.D. Emerging common themes in regulation of pikks and PI3Ks. EMBO J. 2009; 28:3067–3073. 19779456 10.1038/emboj.2009.281 PMC2752028

[32] Matsumoto Y. , Asa A., Modak C., Shimada M. DNA-dependent protein kinase catalytic subunit: the sensor for DNA double-strand breaks structurally and functionally related to Ataxia Telangiectasia mutated. Genes (Basel). 2021; 12:1143. 34440313 10.3390/genes12081143 PMC8394720

[33] Ma Y. , Pannicke U., Lu H., Niewolik D., Schwarz K., Lieber M.R. The DNA-dependent protein kinase catalytic subunit phosphorylation sites in human Artemis. J. Biol. Chem. 2005; 280:33839–33846. 16093244 10.1074/jbc.M507113200

[34] Lees-Miller J.P. , Cobban A., Katsonis P., Bacolla A., Tsutakawa S.E., Hammel M., Meek K., Anderson D.W., Lichtarge O., Tainer J.A. et al. Uncovering DNA-pkcs ancient phylogeny, unique sequence motifs and insights for human disease. Prog. Biophys. Mol. Biol. 2021; 163:87–108. 33035590 10.1016/j.pbiomolbio.2020.09.010 PMC8021618

[35] Liang S. , Thomas S.E., Chaplin A.K., Hardwick S.W., Chirgadze D.Y., Blundell T.L. Structural insights into inhibitor regulation of the DNA repair protein DNA-pkcs. Nature. 2022; 601:643–648. 34987222 10.1038/s41586-021-04274-9 PMC8791830

[36] So S. , Davis A.J., Chen D.J. Autophosphorylation at serine 1981 stabilizes ATM at DNA damage sites. J. Cell Biol. 2009; 187:977–990. 20026654 10.1083/jcb.200906064 PMC2806275

[37] Trakarnphornsombat W. , Kimura H. Live-cell tracking of gamma-H2AX kinetics reveals the distinct modes of ATM and DNA-PK in the immediate response to DNA damage. J. Cell Sci. 2023; 136:jcs260698. 36999484 10.1242/jcs.260698 PMC10163350

[38] Dynan W.S. , Yoo S. Interaction of Ku protein and DNA-dependent protein kinase catalytic subunit with nucleic acids. Nucleic Acids Res. 1998; 26:1551–1559. 9512523 10.1093/nar/26.7.1551 PMC147477

[39] Gottlieb T.M. , Jackson S.P. The DNA-dependent protein kinase: requirement for DNA ends and association with Ku antigen. Cell. 1993; 72:131–142. 8422676 10.1016/0092-8674(93)90057-w

[40] Uematsu N. , Weterings E., Yano K., Morotomi-Yano K., Jakob B., Taucher-Scholz G., Mari P.O., van Gent D.C., Chen B.P., Chen D.J. Autophosphorylation of DNA-PKCS regulates its dynamics at DNA double-strand breaks. J. Cell Biol. 2007; 177:219–229. 17438073 10.1083/jcb.200608077 PMC2064131

[41] Dobbs T.A. , Tainer J.A., Lees-Miller S.P. A structural model for regulation of NHEJ by DNA-pkcs autophosphorylation. DNA Repair (Amst.). 2010; 9:1307–1314. 21030321 10.1016/j.dnarep.2010.09.019 PMC3045832

[42] Jette N. , Lees-Miller S.P. The DNA-dependent protein kinase: a multifunctional protein kinase with roles in DNA double strand break repair and mitosis. Prog. Biophys. Mol. Biol. 2015; 117:194–205. 25550082 10.1016/j.pbiomolbio.2014.12.003 PMC4502593

[43] Gurley K.E. , Kemp C.J. Synthetic lethality between mutation in Atm and DNA-PK(cs) during murine embryogenesis. Curr. Biol. 2001; 11:191–194. 11231155 10.1016/s0960-9822(01)00048-3

[44] Sekiguchi J. , Ferguson D.O., Chen H.T., Yang E.M., Earle J., Frank K., Whitlow S., Gu Y., Xu Y., Nussenzweig A. et al. Genetic interactions between ATM and the nonhomologous end-joining factors in genomic stability and development. Proc. Natl. Acad. Sci. U.S.A. 2001; 98:3243–3248. 11248063 10.1073/pnas.051632098 PMC30638

[45] Neal J.A. , Dang V., Douglas P., Wold M.S., Lees-Miller S.P., Meek K. Inhibition of homologous recombination by DNA-dependent protein kinase requires kinase activity, is titratable, and is modulated by autophosphorylation. Mol. Cell Biol. 2011; 31:1719–1733. 21300785 10.1128/MCB.01298-10 PMC3126343

[46] Chen B.P. , Uematsu N., Kobayashi J., Lerenthal Y., Krempler A., Yajima H., Lobrich M., Shiloh Y., Chen D.J. Ataxia telangiectasia mutated (ATM) is essential for DNA-pkcs phosphorylations at the thr-2609 cluster upon DNA double strand break. J. Biol. Chem. 2007; 282:6582–6587. 17189255 10.1074/jbc.M611605200

[47] Meek K. Activation of DNA-PK by hairpinned DNA ends reveals a stepwise mechanism of kinase activation. Nucleic Acids Res. 2020; 48:9098–9108. 32716029 10.1093/nar/gkaa614 PMC7498359

[48] Lee B.S. , Gapud E.J., Zhang S., Dorsett Y., Bredemeyer A., George R., Callen E., Daniel J.A., Osipovich O., Oltz E.M. et al. Functional intersection of ATM and DNA-dependent protein kinase catalytic subunit in coding end joining during V(D)J recombination. Mol. Cell. Biol. 2013; 33:3568–3579. 23836881 10.1128/MCB.00308-13 PMC3753869

[49] Block W.D. , Yu Y., Merkle D., Gifford J.L., Ding Q., Meek K., Lees-Miller S.P. Autophosphorylation-dependent remodeling of the DNA-dependent protein kinase catalytic subunit regulates ligation of DNA ends. Nucleic Acids Res. 2004; 32:4351–4357. 15314205 10.1093/nar/gkh761 PMC514382

[50] Goodarzi A.A. , Yu Y., Riballo E., Douglas P., Walker S.A., Ye R., Harer C., Marchetti C., Morrice N., Jeggo P.A. et al. DNA-PK autophosphorylation facilitates Artemis endonuclease activity. EMBO J. 2006; 25:3880–3889. 16874298 10.1038/sj.emboj.7601255 PMC1553186

[51] Zhou Y. , Paull T.T. DNA-dependent protein kinase regulates DNA end resection in concert with Mre11-Rad50-Nbs1 (MRN) and ataxia telangiectasia-mutated (ATM). J. Biol. Chem. 2013; 288:37112–37125. 24220101 10.1074/jbc.M113.514398 PMC3873567

[52] Zhou Y. , Lee J.H., Jiang W., Crowe J.L., Zha S., Paull T.T. Regulation of the DNA damage response by DNA-pkcs inhibitory phosphorylation of ATM. Mol. Cell. 2017; 65:91–104. 27939942 10.1016/j.molcel.2016.11.004 PMC5724035

