# Supplementary material for: ATM phosphorylates the FATC domain of DNA-PKcs at threonine 4102 to promote non-homologous end joining
Source: Nucleic Acids Res. 2023 Jun 13;51(13):6770–83. doi: 10.1093/nar/gkad505 (PMC10359628; doi:10.1093/nar/gkad505)
Supplement: gkad505_Supplemental_Files [file gkad505_supplemental_files.zip › Supplementary figures-Rev.pdf]

Accession number	Sequence	Species
hsa_5591	LSEETQVKCLMDQATDPNILGRTWEGWEPWM	Homo sapiens (human)
pps_100981342	LSEETQVKCLMDQATDPNILGRTWEGWEPWM	Pan paniscus (bonobo)
ggo_101128261	LSEETQVKCLMDQATDPNILGRTWEGWEPWM	Gorilla gorilla gorilla (western lowland gorilla)
ptr_464165	LSEETQVKCLMDQATDPNILGRTWEGWEPWM	Pan troglodytes (chimpanzee)
pon_100460398	LSEETQVKCLMDQATDPNILGRTWEGWEPWM	Pongo abelii (Sumatran orangutan)
mcc_708029	LSEETQVKCLIDQATDPNILGRTWEGWEPWM	Macaca mulatta (rhesus monkey)
tge_112630683	LSEETQVKCLIDQATDPNILGRTWEGWEPWM	Theropithecus gelada (gelada)
panu_100999716	LSEETQVKCLIDQATDPNILGRTWEGWEPWM	Papio anubis (olive baboon)
mmur_105885240	LSEETQVRCLIDQATDANILGRTWEGWEPWM	Microcebus murinus (gray mouse lemur)
caty_105592465	LSEETQVKCLIDQATDPNILGRTWEGWEPWM	Cercocebus atys (sooty mangabey)
mni_105496922	LSEETQVKCLIDQATDPNILGRTWEGWEPWM	Macaca nemestrina (pig-tailed macaque)
mcf_102119406	LSEETQVKCLIDQATDPNILGRTWEGWEPWM	Macaca fascicularis (crab-eating macaque)
mthb_126960743	LSEETQVKCLIDQATDPNILGRTWEGWEPWM	Macaca thibetana thibetana (Pere David's macaque)
csab_103236758	LSEETQVKCLIDQATDPNILGRTWEGWEPWM	Chlorocebus sabaeus (green monkey)
rbb_108535350	LSEETQVKCLMDQATDPNILGRTWEGWEPWM	Rhinopithecus bieti (black snub-nosed monkey)
rro_104657634	LSEETQVKCLMDQATDPNILGRTWEGWEPWM	Rhinopithecus roxellana (golden snub-nosed monkey)
tfn_117079536	LSEETQVKCLMDQATDPNILGRTWEGWEPWM	Trachypithecus francoisi (Francois's langur)
pteh_111525257	LSEETQVKCLMDQATDPNILGRTWEGWEPWM	Piliocolobus tephrosceles (Ugandan red Colobus)
cang_105514376	LSEETQVKCLMDQATDPNILGRTWEGWEPWM	Colobus angolensis palliatus (Angola colobus)
leu_105533771	LSEETQVKCLIDQATDPNILGRTWEGWEPWM	Mandrillus leucophaeus (drill)
cimi_108294515	LSEETQVKCLMDQATDPNILGRTWEGWEPWM	Cebus imitator (panamanian white-faced capuchin)
sbq_101053589	LSEETQVKCLIDQATDPNILGRTWEGWEPWM	Saimiri boliviensis boliviensis (Bolivian squirrel monkey)
cjc_100399020	LSEETQVKCLMDQATDPNILGRTWEGWEPWM	Callithrix jacchus (white-tufted-ear marmoset)
pcoq_105824093	LSEETQVRCLIDQATDPNILGRTWEGWEPWM	Propithecus coquereli (coquerel's sifaka)
lcat_123644556	LSEETQVRCLIDQATDPNILGRTWEGWEPWM	Lemur catta (ring-tailed lemur)
csyr_103255463	LSEETQVKCLIDQATDPNILGRTWAGWEPWM	Carlito syrichta (philippine tarsier)
oga_100946687	LSEEMQVKCLIDQATDPNILGRTWEGWEPWM	Otolemur garnettii (small-eared galago)
nle_100586225	LSEEIQVKCLMDQATDPNILGRTWEGWEPWM	Nomascus leucogenys (northern white-cheeked gibbon)
hmh_116813310	LSEEIQVKCLMDQATDPNILGRTWEGWEPWM	Hylobates moloch (silvery gibbon)

Supplementary Figure S1. Conservation of amino acid threonine 4102 (T4102) in DNA-PK$_{cs}$ in primates. BLAST-P analysis were performed using FATC amino acid sequence of human DNA-PK$_{cs}$ in GenomeNet (www.genome.jp), and all primates in the top 100 hits from BLAST-P were further processed for alignment of the FATC domain of DNA-PK$_{cs}$ with CLUSTALW. T4102 is indicated in red.

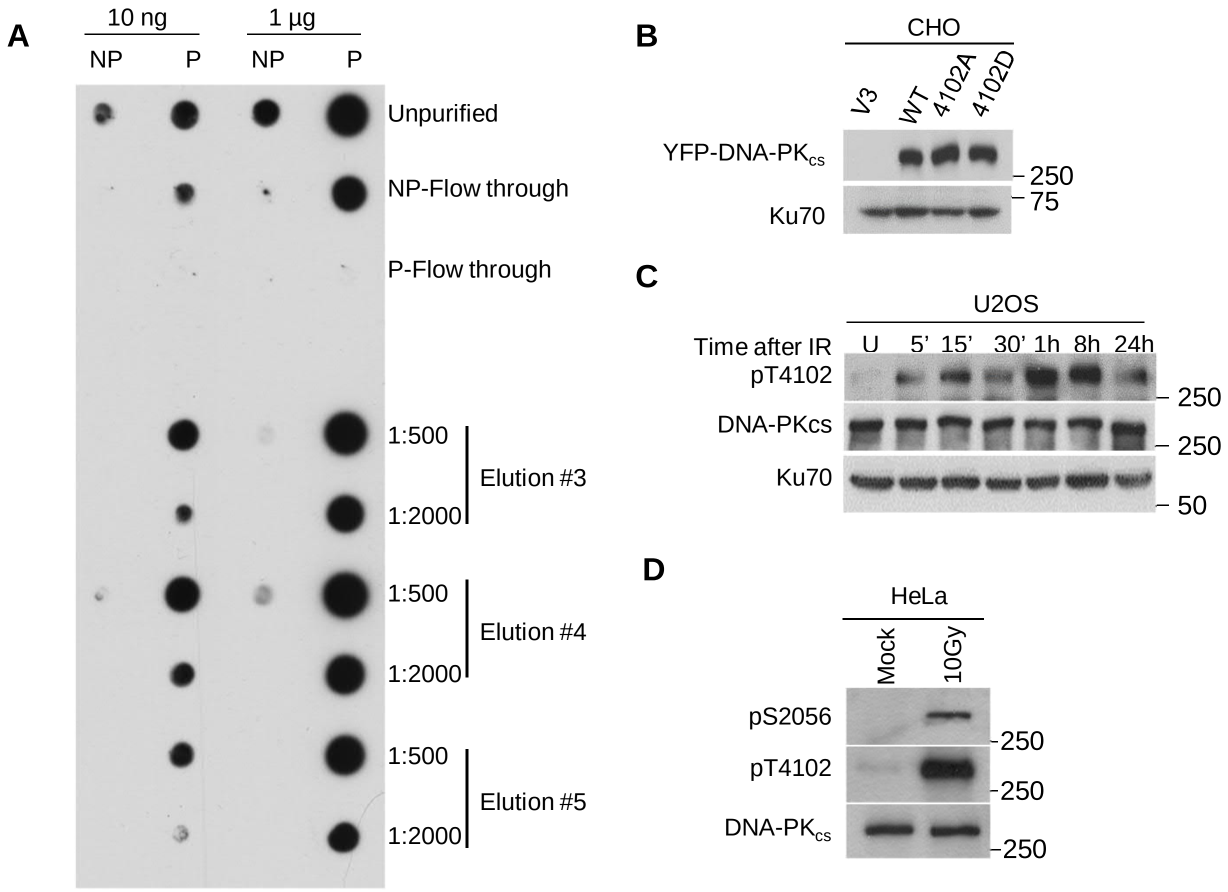

Supplementary Figure S2. DNA-PK$_{cs}$ is phosphorylated at T4102 in response to ionizing radiation (IR) in human cell lines. (A) Verification of DNA-PK$_{cs}$ anti-pT4102 antibody using dot bot analysis. NP, non phosphopeptide; P, T4102 phosphopeptide. 10 ng or 1 µg of NP or P were loaded, transferred to nitrocellulose, and binding of the pT4102 was examined. **(B)** Immunoblotting showing expression of DNA-PK$_{cs}$ in CHO V3 cells and V3 cells stably expressing YFP-tagged DNA-PK$_{cs}$ wild type (WT), phosphorylation-null mutant (T4102A), and phosphorylation-mimic mutant (T4102D). **(C)** Phosphorylation of DNA-PK$_{cs}$ at T4102 at the indicated time following exposure to 10 Gy of IR in the human cell line U2OS. **(D)** Phosphorylation of DNA-PK$_{cs}$ at T4102 30 min post-IR (10 Gy) in the human cell line HeLa.

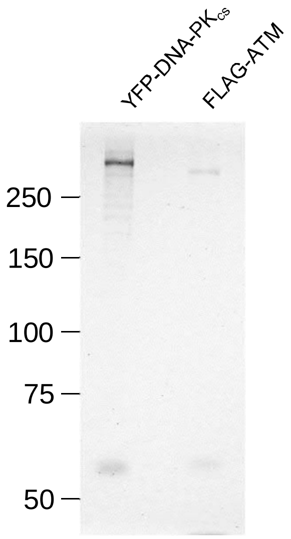

Supplementary Figure S3. Coomassie Blue staining of purified YFP-DNA-PK$_{cs}$ and FLAG-ATM. YFP-tagged DNA-PK$_{cs}$ was purified from CHO V3 cells stably expressing YFP-DNA-PK$_{cs}$. FLAG-ATM was purified from irradiated HT1080 cells stably expressing FLAG-YFP-ATM.

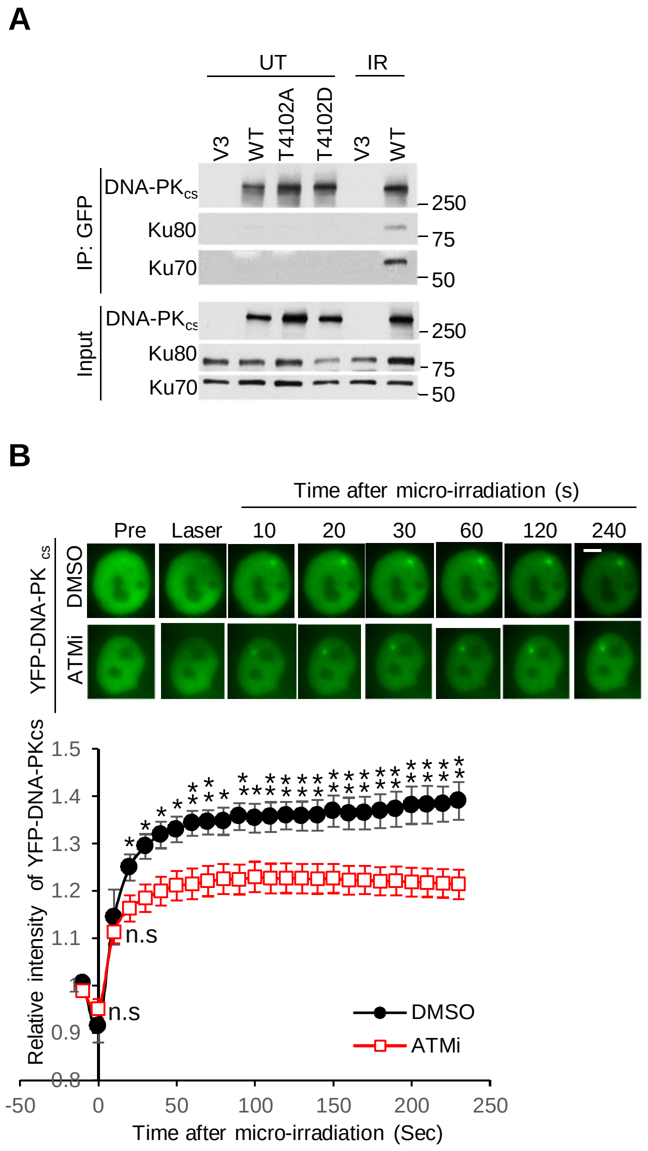

Supplementary Figure S4. (A) Interaction between DNA-PK$_{cs}$ and the Ku heterodimer in the absence of DNA damage. YFP-tagged DNA-PK$_{cs}$ WT, T4102A or T4102D were pulled down from unirradiated CHO V3 cells using GFP antibodies crosslinked to beads. Co-immunoprecipitation of Ku70 and Ku80 with DNA-PK$_{cs}$ was assessed via immunoblotting. Irradiated (10 Gy) V3 cells expressing DNA-PK$_{cs}$ WT was used as a positive control. **(B) Inhibition of ATM kinase attenuates accumulation/retention of DNA-PK$_{cs}$ at laser-induced DSBs.** V3 cells expressing YFP-tagged DNA-PK$_{cs}$ were pretreated with 10 µM ATM inhibitor KU55933 or DMSO for 2 hr and then the recruitment of YFP-DNA-PK$_{cs}$ to laser-induced DSBs was examined. Relative fluorescent intensity of YFP-DNA-PK$_{cs}$ are presented as the mean ± SEM with p-values generated using two-tailed Student T-test. P values: * < 0.05 and ** < 0.01.

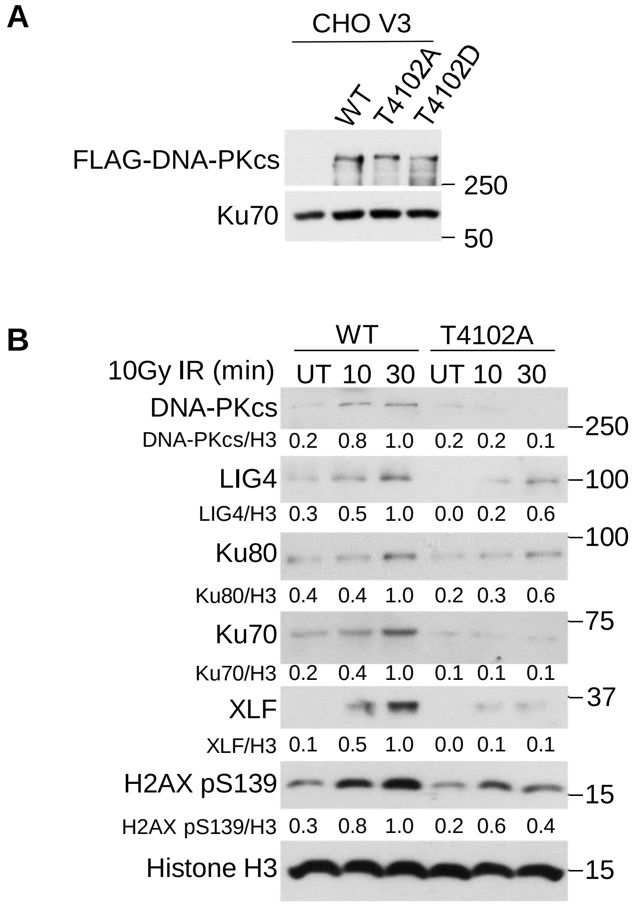

Supplementary Figure S5. Phosphorylation of DNA-PK$_{cs}$ at T4102 promotes the accumulation of the core NHEJ factors at the chromatin fraction following IR. (A) Immunoblotting showing expression of DNA-PK$_{cs}$ in CHO V3 cells and V3 cells stably expressing FLAG-tagged DNA-PK$_{cs}$ wild type (WT), phosphorylation-null mutant (T4102A) and phosphorylation-mimic mutant (T4102D). **(B)** V3 cells complemented with YFP-tagged DNA-PK$_{cs}$ WT or T4102A were mock treated or irradiated with a dose of 10 Gy and allowed to recover for 10 or 30 min. Subsequently, the chromatin fractions were isolated for immunoblotting to examine the recruitment of the proteins listed in the figure to assess IR-induced localization of proteins to the chromatin.

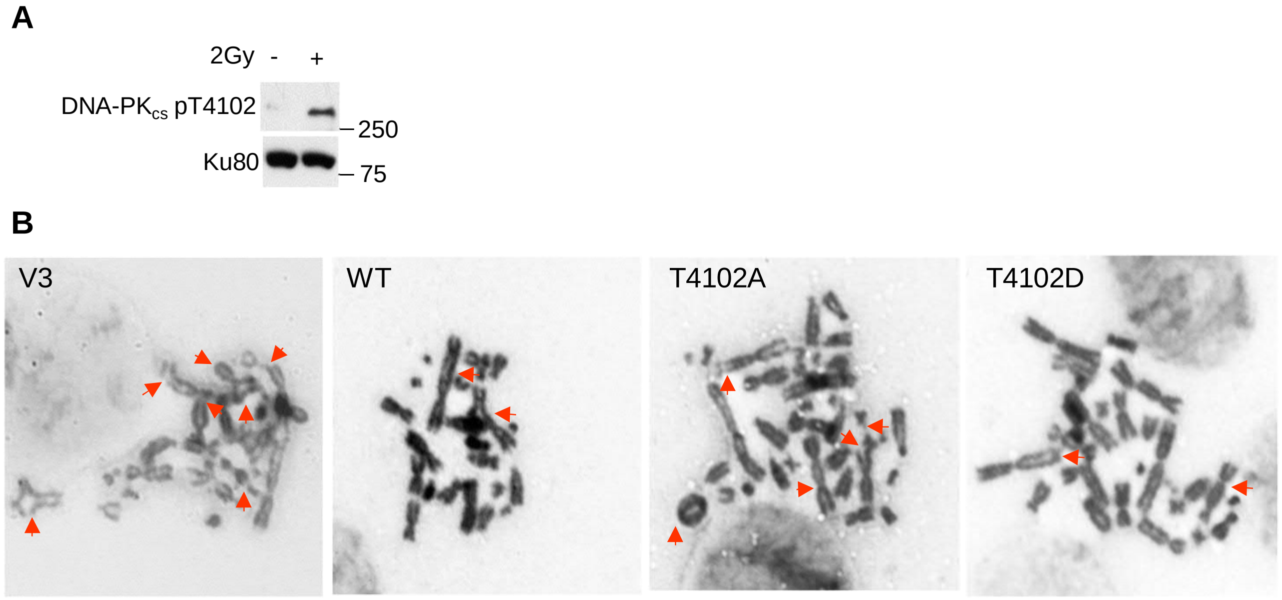

Supplementary Figure S6. Blocking DNA-PK$_{cs}$ T4102 phosphorylation results in increased IR-induced chromosomal aberrations. (A) DNA-PK$_{cs}$ is phosphorylated at T4102 after exposure to a dose of 2 Gy of IR. CHO V3 cells expressing YFP-tagged DNA-PK$_{cs}$ were irradiated with a dose of 2 Gy of IR or mock treated, allowed to recover for 30 min, harvested, processed, and phosphorylation of DNA-PK$_{cs}$ at T4102 was assessed via immunoblotting. Ku80 immunoblottong was used as a loading control. **(B)** Representative images of metaphase spreads of chromosomes from irradiated CHO V3 cells and V3 cells stably expressing YFP-tagged DNA-PK$_{cs}$ WT, T4102A, and T4102D. Abnormal chromosome/chromatin are labeled with red arrows.
