# Supplementary material for: ATM phosphorylates the FATC domain of DNA-PKcs at threonine 4102 to promote non-homologous end joining
Source: Nucleic Acids Res. 2023 Jun 13;51(13):6770–83. doi: 10.1093/nar/gkad505 (PMC10359628; doi:10.1093/nar/gkad505)
Supplement: gkad505_Supplemental_Files [file gkad505_supplemental_files.zip › Supplementary Table S1.pdf]

Table S1 Alignment of DNA-PKcs FATC domain sequences

Accession number	Sequence	Species
**Mammals**		
hsa_5591	LSEETQVKCLMDQATDPNILGRTWEGWEPWM	Homo sapiens (human)
pps_100981342	LSEETQVKCLMDQATDPNILGRTWEGWEPWM	Pan paniscus (bonobo)
ggo_101128261	LSEETQVKCLMDQATDPNILGRTWEGWEPWM	Gorilla gorilla gorilla (western lowland gorilla)
ptr_464165	LSEETQVKCLMDQATDPNILGRTWEGWEPWM	Pan troglodytes (chimpanzee)
pon_100460398	LSEETQVKCLMDQATDPNILGRTWEGWEPWM	Pongo abelii (Sumatran orangutan)
mcc_708029	LSEETQVKCLIDQATDPNILGRTWEGWEPWM	Macaca mulatta (rhesus monkey)
tge_112630683	LSEETQVKCLIDQATDPNILGRTWEGWEPWM	Theropithecus gelada (gelada)
panu_100999716	LSEETQVKCLIDQATDPNILGRTWEGWEPWM	Papio anubis (olive baboon)
mmur_105885240	LSEETQVRCLIDQATDANILGRTWEGWEPWM	Microcebus murinus (gray mouse lemur)
caty_105592465	LSEETQVKCLIDQATDPNILGRTWEGWEPWM	Cercocebus atys (sooty mangabey)
mni_105496922	LSEETQVKCLIDQATDPNILGRTWEGWEPWM	Macaca nemestrina (pig-tailed macaque)
mcf_102119406	LSEETQVKCLIDQATDPNILGRTWEGWEPWM	Macaca fascicularis (crab-eating macaque)
mthb_126960743	LSEETQVKCLIDQATDPNILGRTWEGWEPWM	Macaca thibetana thibetana (Pere David's macaque)
csab_103236758	LSEETQVKCLIDQATDPNILGRTWEGWEPWM	Chlorocebus sabaeus (green monkey)
rbb_108535350	LSEETQVKCLMDQATDPNILGRTWEGWEPWM	Rhinopithecus bieti (black snub-nosed monkey)
rro_104657634	LSEETQVKCLMDQATDPNILGRTWEGWEPWM	Rhinopithecus roxellana (golden snub-nosed monkey)
tfn_117079536	LSEETQVKCLMDQATDPNILGRTWEGWEPWM	Trachypithecus francoisi (Francois's langur)
pteh_111525257	LSEETQVKCLMDQATDPNILGRTWEGWEPWM	Piliocolobus tephrosceles (Ugandan red Colobus)
cang_105514376	LSEETQVKCLMDQATDPNILGRTWEGWEPWM	Colobus angolensis palliatus (Angola colobus)
leu_105533771	LSEETQVKCLIDQATDPNILGRTWEGWEPWM	Mandrillus leucophaeus (drill)
cimi_108294515	LSEETQVKCLMDQATDPNILGRTWEGWEPWM	Cebus imitator (panamanian white-faced capuchin)
sbq_101053589	LSEETQVKCLIDQATDPNILGRTWEGWEPWM	Saimiri boliviensis boliviensis (Bolivian squirrel monkey)
cjc_100399020	LSEETQVKCLMDQATDPNILGRTWEGWEPWM	Callithrix jacchus (white-tufted-ear marmoset)
pcoq_105824093	LSEETQVRCLIDQATDPNILGRTWEGWEPWM	Propithecus coquereli (coquerel's sifaka)
lcat_123644556	LSEETQVRCLIDQATDPNILGRTWEGWEPWM	Lemur catta (ring-tailed lemur)
csyr_103255463	LSEETQVKCLIDQATDPNILGRTWAGWEPWM	Carlito syrichta (philippine tarsier)
oga_100946687	LSEEMQVKCLIDQATDPNILGRTWEGWEPWM	Otolemur garnettii (small-eared galago)
nle_100586225	LSEEIQVKCLMDQATDPNILGRTWEGWEPWM	Nomascus leucogenys (northern white-cheeked gibbon)
hmh_116813310	LSEEIQVKCLMDQATDPNILGRTWEGWEPWM	Hylobates moloch (silvery gibbon)
shr_100915132	LSEETQVKCLMDQATDPNVLGRTWIGWEPWM	Sarcophilus harrisii (Tasmanian devil)
cpoc_100724826	LSVETQVRCLLDQATDPNILGRTWEGWEPWM	Cavia porcellus (domestic guinea pig)
ssc_100156379	LSEEVQVKCLIDQATDPNILGRTWEGWEPWM	Sus scrofa (pig)
pcw_110212506	LSEETQVKCLMDQATDPNVLGRTWTGWEPWM	Phascolarctos cinereus (koala)
etf_101661361	LSEEIQVKCLIDQATDPNILGRTWEGWEPWM	Echinops telfairi (small Madagascar hedgehog)
biu_109568449	LSEEAQVRCLIDQATDPNILGRTWEGWEPWM	Bos indicus (zebu cattle)
btax_128059433	LSEEAQVRCLVDQATDPNILGRTWEGWEPWM	Budorcas taxicolor (takin)
bta_512740	LSEEAQVRCLIDQATDPNILGRTWEGWEPWM	Bos taurus (cow)
bbis_104987853	LSEEAQVRCLIDQATDPNILGRTWEGWEPWM	Bison bison bison (American bison)
bbub_102402820	LSEEAQVRCLIDQATDPNILGRTWEGWEPWM	Bubalus bubalis (water buffalo)
bom_102285992	LSEEAQVRCLIDQATDPNILGRTWEGWEPWM	Bos mutus (wild yak)
chx_102171625	LSEEAQVRCLVDQATDPNILGRTWEGWEPWM	Capra hircus (goat)
ccan_109688672	LSEEAQVKCLMDQATDPNILGRTWEGWEPWM	Castor canadensis (American beaver)
nvs_122904613	LSEEAQVKCLIDQATDPNILGRTWQGWEPWM	Neogale vison (American mink)
llv_125096736	LSEEAQVKCLIDQATDPNILGRTWQGWEPWM	Lutra lutra (Eurasian river otter)

mpuf_101687340	LSEEAQVKCLIDQATDPNILGRTWQGWEPWM	Mustela putorius furo (domestic ferret)
cbai_105063642	LSEEAQVKCLIDQATDPNILGRTWEGWEPWM	Camelus bactrianus (Bactrian camel)
cfr_102519846	LSEEAQVKCLIDQATDPNILGRTWEGWEPWM	Camelus ferus (Wild Bactrian camel)
cdk_105095924	LSEEAQVKCLIDQATDPNILGRTWEGWEPWM	Camelus dromedarius (Arabian camel)
vpc_102542127	LSEEAQVKCLIDQATDPNILGRTWEGWEPWM	Vicugna pacos (alpaca)

nasi_112398224	LSEEAQVKCLIDQATDPNILGRTWEGWEPWM	Neophocaena asiaeorientalis asiaeorientalis (Yangtze finless porpoise)
psiu_116742151	LSEEAQVKCLIDQATDPNILGRTWEGWEPWM	Phocoena sinus (vaquita)
dle_111165833	LSEEAQVKCLIDQATDPNILGRTWEGWEPWM	Delphinapterus leucas (beluga whale)
bacu_103019606	LSEEAQVKCLIDQATDPNILGRTWEGWEPWM	Balaenoptera acutorostrata scammoni (minke whale)
lve_103090600	LSEEAQVKCLIDQATDPNILGRTWEGWEPWM	Lipotes vexillifer (Yangtze River dolphin)
pcad_102996579	LSEEAQVKCLIDQATDPNILGRTWEGWEPWM	Physeter catodon (sperm whale)

cge_100770748	LSEETQVKCLVDQATDPNILGRTWEGWEPWM	Cricetulus griseus (Chinese hamster)
prob_127226869	LSEETQVKCLVDQATDPNILGRTWEGWEPWM	Phodopus roborovskii (desert hamster)
maua_101824721	LSEETQVKCLVDQATDPNILGRTWEGWEPWM	Mesocricetus auratus (golden hamster)
morg_121464862	LSEETQVKCLVDQATDPNILGRTWEGWEPWI	Microtus oregoni (creeping vole)
mfot_126490314	LSEETQVKCLVDQATDPNILGRTWEGWEPWI	Microtus fortis (reed vole)
aamp_119824783	LSEETQVKCLVDQATDPNILGRTWEGWEPWI	Arvicola amphibius (Eurasian water vole)
tod_119241502	LSEEAQVKCLIDQATDPNILGRTWEGWEPWM	Talpa occidentalis (Iberian mole)
mmu_19090	LSEETQVKCLVDQATDPNILGRTWEGWEPWM	Mus musculus (house mouse)
mcal_110311468	LSEETQVKCLVDQATDPNILGRTWEGWEPWM	Mus caroli (Ryukyu mouse)
mpah_110330004	LSEETQVKCLVDQATDPNILGRTWEGWEPWM	Mus pahari (shrew mouse)
pleu_114709460	LSEETQVKCLVDQATDPNILGRTWEGWHPWM	Peromyscus leucopus (white-footed mouse)
mcoc_116085621	LSEETQVKCLVDQATDPNILGRTWQGWEPWM	Mastomys coucha (southern multimammate mouse)
rno_360748	LSEETQVKCLVDQATDPNILGRTWEGWEPWM	Rattus norvegicus (rat)
dsp_122113064	LSEETQVKCLMDQATDPNILGRTWEGWEPWM	Dipodomys spectabilis (banner-tailed kangaroo rat)
dord_105980395	LSEETQVKCLMDQATDPNILGRTWEGWEPWM	Dipodomys ordii (Ord's kangaroo rat)
mun_110561334	LSEETQVKCLVDQATDPNILGRTWEGWDPWM	Meriones unguiculatus (Mongolian gerbil)
ncar_124981191	LSEETQVKCLLDQATDPNILGRTWEGWEPWM	Sciurus carolinensis (gray squirrel)
opi_101519750	LSAETQVKCLIDQATDPNVLGRTWAGWEPWM	Ochotona princeps (American pika)
afz_127543891	LSEETQVKCLMDQATDPNVLGRTWIGWEPWM	Antechinus flavipes (yellow-footed antechinus)

rfq_117033631	LSEEAQVKCLIDQATDPNILGRTWEGWEPWM	Rhinolophus ferrumequinum (greater horseshoe bat)
hai_109392680	LSEEAQVKCLIDQATDPNILGRTWEGWEPWM	Hipposideros armiger (great roundleaf bat)
pvp_105309245	LSEEAQVKCLIDQATDPNILGRTWIGWEPWM	Pteropus vampyrus (large flying fox)
pgig_120592708	LSEEAQVKCLIDQATDPNILGRTWIGWEPWM	Pteropus giganteus (Indian flying fox)
pale_102895801	LSEEAQVKCLIDQATDPNILGRTWIGWEPWM	Pteropus alecto (black flying fox)
ray_107505079	LSEEAQVKCLIDQATDPNILGRTWIGWEPWM	Rousettus aegyptiacus (Egyptian rousette)
myd_102770810	LSEEAQVKCLIDQATDPNILGRTWEGWEPWV	Myotis davidii (David's myotis)
myb_102238664	LSEEAQVKCLIDQATDPNILGRTWEGWEPWM	Myotis brandtii (Brandt's bat)
mlf_102419064	LSEEAQVKCLIDQATDPNILGRTWEGWEPWM	Myotis lucifugus (little brown bat)
pkl_118718434	LSEEAQVKCLIDQATDPNILGRTWEGWEPWM	Pipistrellus kuhlii (Kuhl's pipistrelle)

Birds

dpub_104303091	LSEETQVRCLIDQATDPNILGRVWEGWEPWM	Dryobates pubescens (Downy woodpecker)
arow_112965430	LSEETQVKCLIDQATDPNVLGRVWEGWEPWM	Apteryx rowi (Okarito brown kiwi)

aam_106494392	LSEETQVKCLIDQATDPNVLGRVWEGWEPWM	Apteryx mantelli mantelli (North Island brown kiwi)
scam_104149477	LSEETQVKCLIDQATDPNILGRAWEGWEPWM	Struthio camelus australis (South African ostrich)
hald_104317668	LSEETQVRCLIDQATDPNVLGRVWEGWEPWM	Haliaeetus albicilla (white-tailed eagle)
gcl_127012553	LSEETQVRCLIDQATDPNILGRVWEGWEPWM	Gymnogyps californianus (California condor)
fpg_101914535	LSEETQVRCLIDQATDPNILGRVWEGWEPWM	Falco peregrinus (peregrine falcon)
afor_103904238	LSEETQVRCLIDQATDPNILGRVWEGWEPWM	Aptenodytes forsteri (emperor penguin)
padl_103923190	LSEETQVRCLIDQATDPNILGRVWEGWEPWM	Pygoscelis adeliae (Adelie penguin)
dne_112984824	LSEETQVKCLIDQATDPNILGRVWEGWEPWM	Dromaius novaehollandiae (emu)
fga_104081917	LSEETQVRCLIDQATDPNILGRVWEGWEPWM	Fulmarus glacialis (Northern fulmar)
brhi_104493648	LSEETQVRCLIDQATDPNVLGRTWEGWEPWM	Buceros rhinoceros silvestris (Rhinoceros hornbill)
svg_106851955	LSEETQVRCLIDQATDPNILGRVWEGWEPWM	Sturnus vulgaris (common starling)
fab_101819416	LSEETQVRCLIDQATDPNILGRVWEGWEPWM	Ficedula albicollis (collared flycatcher)
cbrc_103625526	LSEETQVRCLIDQATDPNILGRVWEGWEPWM	Corvus brachyrhynchos (American crow)
ccw_104690434	LSEETQVRCLIDQATDPNILGRVWEGWEPWM	Corvus cornix (hooded crow)
pmaj_107200430	LSEETQVRCLIDQATDPNILGRVWEGWEPWM	Parus major (Great Tit)
phi_102108488	LSEETQVRCLIDQATDPNILGRVWEGWEPWM	Pseudopodoces humilis (Tibetan ground-tit)
ccae_111924874	LSEETQVRCLIDQATDPNILGRVWEGWEPWM	Cyanistes caeruleus (blue tit)
pruf_121356858	LSEETQVRCLIDQATDPNILGRVWEGWEPWM	Pyrgilauda ruficollis (rufous-necked snowfinch)
otc_121340193	LSEETQVRCLIDQATDPNILGRVWEGWEPWM	Onychostruthus taczanowskii (white-rumped snowfinch)
scan_103827354	LSEETQVRCLIDQATDPNILGRVWEGWEPWM	Serinus canaria (common canary)
zab_102066177	LSEETQVRCLIDQATDPNILGRVWEGWEPWM	Zonotrichia albicollis (white-throated sparrow)
etl_114060600	LSEETQVRCLIDQATDPNILGRVWEGWEPWM	Empidonax traillii (willow flycatcher)

Reptiles

hcg_128324456	LSEETQVKCLIDQATDPNILGRVWEGWEPWM	Hemicordylus capensis (graceful crag lizard)
sund_121929123	LSEETQVKCLIDQATDPNILGRVWEGWEPWI	Sceloporus undulatus (fence lizard)
pvt_110077653	LSEETQVKCLIDQATDPNILGRVWEGWEPWM	Pogona vitticeps (central bearded dragon)
ctig_120303854	LTEEIQVKCLIDQATDPNILGRTWEGWEPWM	Crotalus tigris (Tiger rattlesnake)
acs_100562493	LSEEIQVKCLIDQATDPNILGRVWEGWEPWM	Anolis carolinensis (green anole)
tsr_106554042	LAEEIQVKCLIDQATDPNILGRTWEGWEPWM	Thamnophis sirtalis (common garter snake)
pgut_117672768	LAEEIQVKCLIDQATDPNILGRTWQGWEPWM	Pantherophis guttatus (corn snake):
pmur_107287153	LTEEIQVKCLIDQATDPNILGRTWEGWEPWM	Protobothrops mucrosquamatus (Taiwan habu)
pss_102454928	LSEEIQVKCLLDQATDPNLLGRVWEGWEPWM	Pelodiscus sinensis (Chinese soft-shelled turtle)
cmy_102937565	LLEEIQVKCLIDQATDPNLLGRVWEGWEPWM	Chelonia mydas (green sea turtle)
ctic_101936824	LSEEIQVRCLLDQATDPNLLGRVWEGWEPWM	Chrysemys picta (western painted turtle)
tst_117873465	LSEEIQVRCLLDQATDPNLLGRVWEGWEPWM	Trachemys scripta elegans (red-eared slider)
cabi_116836465	LLEEIQVRCLIDQATDPNLLGRVWEGWEPWM	Chelonoidis abingdonii (Abingdon island giant tortoise)
mrv_120399262	LLEEIQVRCLIDQATDPNLLGRVWEGWEPWM	Mauremys reevesii (Reeves's turtle)
pbi_103059879	LTEEVQVKCLIDQATDPNILGRAWEGWEPWM	Python bivittatus (Burmese python)
vko_123020429	LSEEAQVKCLIDQATDPNILGRVWEGWESWM	Varanus komodoensis (Komodo dragon)
puma_114600632	LSEETQVKCLIDQARDPNVLGRVWEGWEPWM	Podarcis muralis (common wall lizard)
zvi_118089778	LSEETQVKCLIDQARDPNVLGRVWEGWEPWM	Zootoca vivipara (common lizard)
gja_107122359	LSEEIQVKCLIDQATDSNILGRVWEGWEPWM	Gekko japonicus (Schlegel's Japanese gecko)
stow_125438905	LSEEMQVKCLIDQATDPNILGRVWEGWEPWM	Sphaerodactylus townsendi (Townsend's least gecko)
cpoo_109314867	LTEETQVKCLIDQATDPNILGRVWAGWESWM	Crocodylus porosus (Australian saltwater crocodile)

asn_102387673	LTEETQVKCLIDQATDPNILGRVWAGWESWM	Alligator sinensis (Chinese alligator)
amj_102575311	LTEETQVKCLIDQATDPNILGRVWAGWESWM	Alligator mississippiensis (American alligator)
ggn_109298708	LTEETQVKCLIDQATDPNILGRVWAGWESWM	Gavialis gangeticus (Gharial)

Amphibians

xla_373602	LTEETQVQCLIDQATDPNILGRVWKGWEPWI	Xenopus laevis (African clawed frog)
xtr_100488663	LSEEAQVQCLIDQATDPNILGRVWKGWEPWI	Xenopus tropicalis (tropical clawed frog)
npr_108792226	LSEETQVQCLIDQATDPNILGRAWKGWEPWI	Nanorana parkeri (Xizang Plateau frog)
rtem_120940971	LSEEAQVQCLIDQATDPNILGRTWRGWEPWI	Rana temporaria (common frog)
bbuf_121002127	LSEEIQVKCLIDQATDPNILGRVWTGWEPWM	Bufo bufo (common toad)
bgar_122938497	LSEEIQVKCLIDQATDPNILGRVWIGWEPWM	Bufo gargarizans (Asiatic toad)
XP_029447109.1	LTEETQVKCLLDQATDPNILGRAWQQWEPWM	Rhinatrema bivittatum (two-lined caecilian)

Fish

Lcm_102352949	LPVETQVACLIDQATDPNILGRVWEGWEPWM	Latimeria chalumnae (coelacanth)
Arut_117435350	LSVETQVECLIDQATDPNILGRVWVGWESWV	Acipenser ruthenus (sterlet)
Pspa_121314412	LSVETQVECLIDQATDPNILGRVWAGWEPWV	Polyodon spathula (Mississippi paddlefish)
dre_562283	LTVEDQVDCLLDQATDPNILGRVWIGWEPWI	Danio rerio (zebrafish)
srx_107724397	LSVEDQVDCLLDQATDPNILGRVWIGWEPWI	Sinocyclocheilus rhinocerous
sanh_107684509	LSVEDQVDCLLDQATDPNILGRVWIGWEPWI	Sinocyclocheilus anshuiensis
sgh_107591017	LSVEDQVDCLLDQATDPNILGRVWIGWEPWI	Sinocyclocheilus grahami (golden-line barbel)
ccar_109092833	LSVEDQVDCLLDQATDPNILGRVWIGWKPWI	Cyprinus carpio (common carp)
caua_113106164	LSVEDQVDCLLDQATDPNILGRVWIGWEPWI	Carassius auratus (goldfish)
cgib_127962738	LSVEDQVDCLLDQATDPNILGRVWIGWEPWI	Carassius gibelio (silver crucian carp)
pprm_120493685	LSVEDQVDCLLDQATDPNILGRVWVGWEPWV	Pimephales promelas (fathead minnow)
mamb_125277589	LSVEDQVDCLLDQATDPNILGRVWAGWEPWI	Megalobrama amblycephala (Wuchang bream)
cide_127515514	LSVEDQVDCLLDQATDPNILGRVWVGWEPWI	Ctenopharyngodon idella (grass carp)
masi_127451564	LSVEDQVDCLLDQATDPNILGRVWIGWEPWI	Myxocyprinus asiaticus (Chinese sucker)
ipu_108268160	LSVEEQVDCLIDQATDPNILGRTFQGWEAWM	Ictalurus punctatus (channel catfish)
phyp_113540485	LSVEEQVDCLIDQATDPNILGRTYTGWEPWM	Pangasianodon hypophthalmus (striped catfish)
smeo_124386883	LSVEEQVNCLIDQATDANILGRTFTGWEPWM	Silurus meridionalis (southern catfish)
tfd_113657371	LSVEEQVDCLIDQATDPNILGRTFVGWEPWM	Tachysurus fulvidraco (yellow catfish)
amex_103028565	LSVEHQVDCLIDQATDANLLGRVWVGWEPWM	Astyanax mexicanus (Mexican tetra)
eee_113583195	LSVDEQVDCLIDQATDANLLGRAWIGWEPWI	Electrophorus electricus (electric eel)
tru_101073073	LSVEDQVRCLLDQAMDPNVLGRVWVGWDPWM	Takifugu rubripes (torafugu)
tng:GSTEN00005152G001	LSVDKQVDCLLDQAMDPNVLGRVWAGWEPWM	Tetraodon nigroviridis (spotted green pufferfish)
lco_104928964	LSVEKQVDCLLDQAMDPNVLGRVWAGWEPWF	Larimichthys crocea (large yellow croaker)
cgob_115025495	LSVEKQVDCLLDQAMDPNVLGRVWVGWEPWI	Cottoperca gobio
ely_117264763	LPVEKQVDCLLDQAMDPNVLGRVWAGWEPWF	Epinephelus lanceolatus (giant grouper)
efo_125881735	LPVEKQVDCLLDQAMDPNVLGRVWAGWEPWF	Epinephelus fuscoguttatus (brown-marbled grouper)
plep_121960509	LSVEKQVDCLLDQAMDPNVLGRVWAGWEPWL	Plectropomus leopardus (leopard coral grouper)
sluc_116055758	LPVEKQVDCLLDQAMDPNVLGRVWVGWEPWM	Sander lucioperca (pikeperch)
ecra_117938236	LPVEKQVDCLLDQAMDPNVLGRVWVGWEPWM	Etheostoma cragini (Arkansas darter)
esp_116685823	LPVEKQVDCLLDQAMDPNVLGRVWVGWEPWM	Etheostoma spectabile (orangethroat darter)

pflv_114548975	LPVEKQVDCLLDQAMDPNVLGRVWAGWEPWM	Perca flavescens (yellow perch)
gat_120811646	LSVEKQVDCLLDQAMDPNVLGRVWVGWEPWF	Gasterosteus aculeatus (three-spined stickleback)
ppug_119198541	LSVEKQVDCLLDQAMDPNVLGRVWVGWEPWF	Pungitius pungitius (ninespine stickleback)
msam_119907076	LSVENQVDCLLDQAMDPNVLGRVWAGWEPWF	Micropterus salmoides (largemouth bass)
schu_122866791	LSVEKQVDCLLDQAMDPNVLGRVWVGWEPWL	Siniperca chuatsi (mandarin fish)
cud_121527198	LTVEQQVDCLLDQAMDPNVLGRVWVGWEPWV	Cheilinus undulatus (humphead wrasse)
alat_119008696	LSVEKQVDCLLDQAMDPNVLGRVWVGWEPWV	Acanthopagrus latus (yellowfin seabream)
mze_101484851	LSVENQVDCLLDQAMDPNVLCRVWVGWEPWI	Maylandia zebra (zebra mbuna)
onl_100706243	LSVENQVDCLLDQAMDPNVLSRVWVGWEPWI	Oreochromis niloticus (Nile tilapia)
oau_116335764	LSVENQVDCLLDQAMDPNVLSRVWVGWEPWI	Oreochromis aureus (blue tilapia)
ola_101171206	LSVEKQVECLLDHATDPNVLGRVYVGWEPWI	Oryzias latipes (Japanese medaka)
oml_112151428	LSVEKQVDCLLDQAMDPNVLGRVWHGWEPWI	Oryzias melastigma (Indian medaka)
xma_102221760	LSVEQQVDCLLDQAMDPNVLGRVYAGWEPWI	Xiphophorus maculatus (southern platyfish)
xco_114137103	LSVEQQVDCLLDQAMDPNVLGRVYAGWEPWI	Xiphophorus couchianus (Monterrey platyfish)
xhe_116711736	LSVEQQVDCLLDQAMDPNVLGRVYAGWEPWI	Xiphophorus hellerii (green swordtail)
pret_103482327	LSVEQQVDCLLDQAMDPNVLGRVYAGWEPWI	Poecilia reticulata (guppy)
pfor_103143342	LSVEQQVDCLLDQAMDPNVLGRVYAGWEPWI	Poecilia formosa (Amazon molly)
plai_106958239	LSVEQQVDCLLDQAMDPNVLGRVYVGWEPWI	Poecilia latipinna (sailfin molly)
pmei_106914554	LSVEQQVDCLLDQAMDPNVLGRVYAGWEPWI	Poecilia mexicana (shortfin molly)
gaf_122824240	LSVEQQVDCLLDQAMDPNVLGRVYAGWEPWI	Gambusia affinis (western mosquitofish)
cvg_107084919	LTVEQQVECLLDQATDPNVLGRVWVGWEPWF	Cyprinodon variegatus (sheepshead minnow)
ctul_119795987	LTVEQQVECLLDQATDPNVLGRVWGGWEPWF	Cyprinodon tularosa (White Sands pupfish)
gmu_124858125	LSVEQQVDCLLDQAMDPNVLSRVWVGWESWI	Girardinichthys multiradiatus (darkedged splitfin)
nfu_107382641	LSVEKQVDCLLDQAMDPNVLGRVYAGWESWM	Nothobranchius furzeri (turquoise killifish)
kmr_108235234	LSVEKQVDCLLDQATDPNVLGRAWMGWEPWL	Kryptolebias marmoratus (mangrove rivulus)
alim_106517127	LSVEKQVDCLLDQATDPNVLGRVWVGWEPWF	Austrofundulus limnaeus (annual killifish)
nwh_119426588	LSVEKQVDCLLDQAMDPNVLGRVWAGWEPWF	Nematolebias whitei (Rio pearlfish)
aoce_111574562	LSVEKQVDCLLDQAMDPNVLGRVWVGWEPWI	Amphiprion ocellaris (clown anemonefish)
mcep_124995515	LSVEKQVDCLLDQAMDPNILGRVWAGWEPWI	Mugil cephalus (flathead mullet)
csem_103377075	LSVEKQVDCLIDQATDPNVLGRVWAGWEPGF	Cynoglossus semilaevis (tongue sole)
pov_109629764	LSAEQQVDCLLDQAMDPNVLGRVWVGWESWF	Paralichthys olivaceus (Japanese flounder)
ssen_122771322	LSVEKQVDCLIDQATDPNVLGRVWAGWEPWF	Solea senegalensis (Senegalese sole)
hhip_117754282	LSAEKQVECLLDQAMDPNVLGRVWVGWESWF	Hippoglossus hippoglossus (Atlantic halibut)
hsp_118098382	LSAEKQVECLLDQAMDPNVLGRVWVGWESWF	Hippoglossus stenolepis (Pacific halibut)
lcf_108898307	LSVEKQVDCLLDQAMDPNVLARVWVGWEPWI	Lates calcarifer (barramundi perch)
sdu_111228243	LSVEKQVDCLLDQAMDPNVLGRVWGGWEPWI	Seriola dumerili (greater amberjack)
slal_111648260	LSVEKQVDCLLDQAMDPNVLGRVWVGWEPWI	Seriola lalandi dorsalis (Yellowtail amberjack)
xgl_120789644	LSVEKQVDCLLDQATDPNVLGRVWEGWQPWF	Xiphias gladius (swordfish)
hcq_109507813	LSVEKQVECLLDQATDANVLGRTWAGWEPWF	Hippocampus comes (tiger tail seahorse)
sscv_125985671	LSVEKQVECLLDQATDANVLGRVYAGWEPWI	Syngnathus scovelli (Gulf pipefish)
bpec_110165311	LLVEKQVDCLLDQAMDPNILGRVWAGWEPWV	Boleophthalmus pectinirostris (great blue-spotted mudskipper)
malb_109952212	LSVERQVDCLLDQAMDPNVLGRTFAGWEPWM	Monopterus albus (swamp eel)
bspl_114853392	LSVEQQVNCLLDQAMDPNLLGRVYAGWEPWV	Betta splendens (Siamese fighting fish)
sasa_106579558	LSVESQVDCLLDQAMDPNILGRVWVGWEPWI	Salmo salar (Atlantic salmon)
stru_115150674	LSVESQVDCLLDQAMDPNILGRVWVGWEPWI	Salmo trutta (river trout)
otw_112215359	LSVESQVDCLLDQAMDPNILGRVWVGWEPWI	Oncorhynchus tshawytscha (Chinook salmon)

omy_110535300	LSVE**S**QVDCLLDQAMDPNILGRVWVGWEPWI	Oncorhynchus mykiss (rainbow trout)
ogo_123991347	LSVE**S**QVDCLLDQAMDPNILGRVWVGWEPWI	Oncorhynchus gorbuscha (pink salmon)
one_115104906	LSVE**S**QVDCLLDQAMDPNILGRVWVGWEPWI	Oncorhynchus nerka (sockeye salmon)
salp_111972800	LSVE**S**QVDCLLDQAMDPNILGRVWVGWEPWI	Salvelinus sp. IW2-2015 (Arctic char)
snh_120027880	LSVE**S**QVDCLLDQAMDPNILGRVWVGWEPWI	Salvelinus namaycush (lake trout)
cclu_121549875	LSVE**N**QVDCLLDQAMDPNILGRVWMGWEPWI	Coregonus clupeaformis (lake whitefish)
els_105019560	LSVE**S**QVECLLDQAMDPNILGRVWAGWEPWI	Esox lucius (northern pike)
sfm_108919710	LSVE**T**QVDCLLDQATDPNILGRVWIGWEAWI	Scleropages formosus (Asian bonytongue)
pki_111854631	LSVE**T**QVDCLLDQATDPNILGRVWNGWEPWI	Paramormyrops kingsleyae
aang_118209591	LSVE**A**QVDCLLDQATDPNVLGRVWIGWEPWM	Anguilla anguilla (European eel)
loc_102698219	LAVE**I**QVECLLDQATDPNILGRVYMGWEPWI	Lepisosteus oculatus (spotted gar)
pspa_121314412	LSVE**T**QVECLIDQATDPNILGRVWAGWEPWV	Polyodon spathula (Mississippi paddlefish)
arut_117435350	LSVE**T**QVECLIDQATDPNILGRVWVGWESWV	Acipenser ruthenus (sterlet)
psex_120530210	LSVE**T**QVECLIDQATDPNLLGRVWQGWEPWV	Polypterus senegalus (gray bichir)
lcm_102352949	LPVE**T**QVACLIDQATDPNILGRVWEGWEPWM	Latimeria chalumnae (coelacanth)

Notes:
FATC domain of human DNA-PKcs with sequence "LSEETQVKCLMDQATDPNILGRTWEGWEPWM" was submitted to GenomeNet (www.genome.jp) to perform BLAST-P. FATC domain sequences of mammals and birds were aligned by CLUSTALW within the Top100 hits from the resulting BLASTP hits. For reptiles, Amphibians and fishes, the alignment was performed with sequence from Top 500 hits.
